# Precise and rapid release of X-ray activated autophagy inhibitors for radiotherapy sensitization of hypopharyngeal carcinoma

**DOI:** 10.1016/j.mtbio.2026.103070

**Published:** 2026-03-25

**Authors:** Xinyuan Luo, Rui Rong, Jiao Zhou, Qiongling Huang, Yanjun Huang, Xiaofang Chen, Ming Lu, Shenjiong Ruan, Kehui Chen, Yuanchang Zhou, Zexin Li, Jiwang Zhang, Yun Zhang, Chaohui Zheng

**Affiliations:** a Department of Otolaryngology, the Second Affiliated Hospital, Fujian Medical University, Quanzhou, Fujian, 362000, China; b State Key Laboratory of Structural Chemistry, Fujian Institute of Research on the Structure of Matter, Chinese Academy of Sciences, Fuzhou, Fujian, 350002, China; c Xiamen Key Laboratory of Rare Earth Photoelectric Functional Materials, Xiamen Institute of Rare Earth Materials, Xiamen, Fujian, 361021, China; d First Affiliated Hospital of Xiamen University, School of Medicine, Xiamen University, Xiamen, 361003, China

**Keywords:** Precise release, Rapid release, Autophagy inhibitor, Radiotherapy resistance, Radiosensitization

## Abstract

Protective autophagy represents a major factor responsible for radiotherapy resistance in tumor cells, leading to therapeutic failure, metastasis, and recurrence. Autophagy inhibitors can enhance radiosensitivity by suppressing protective autophagy in tumor. However, the usage of autophagy inhibitors is limited by the issues including non-specific release and delayed delivery. In this work, we develop an X-ray-activatable nanocarrier ZGGC@MON-CQ (ZMC) for precise and rapid release of autophagy inhibitors in tumor cells which enables significantly enhanced radiosensitization. The designed ZMC can precisely probe tumors via background-free fluorescence under the guidance of afterglow of high signal-to-noise ratio 50.7 which specifically enhanced by the tumor microenvironment (TME). Under X-ray irradiation, the tetrasulfide-incorporated mesoporous silica coating on the surface of afterglow nanoparticle undergoes cleavage, leading to rapid release of the autophagy inhibitors (50% released within 20 min). The efficient release is facilitated by a highly active redox-cleavage reaction between the tetrasulfide bonds and the abundant H_2_O_2_ generated by X-ray excitation in the mildly acidic condition of TME. In animal models, the ZMC exhibit highly effective radiosensitization in hypopharyngeal carcinoma that is highly dependent on radiotherapy. This work overcomes the limitations of conventional drug delivery systems, such as off-target release and slow response (e.g., glutathione (GSH)-triggered release exceeding 6 h), and offers a new strategy for precise and rapid delivery of autophagy inhibitors.

## Graphical abstract

**Figure:**
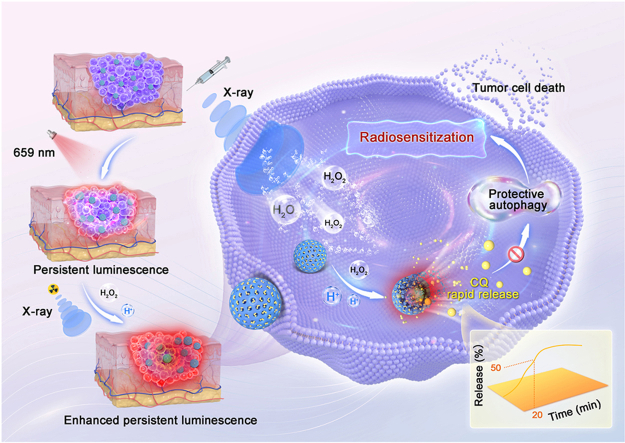

## 1 Introduction

Hypopharyngeal carcinoma (HPC) is the malignancy with the poorest prognosis among head and neck squamous cell carcinomas, with a five-year survival rate of only 25-35% [1,2]. The hypopharynx is a vital organ responsible for respiration, swallowing, and vocalization. For the treatment of HPC, the preservation of organ function is as important as the improvement of survival rate [3]. HPC as poorly differentiated squamous cell carcinoma that exhibits high sensitivity to radiotherapy. Therefore, radiotherapy is a key approach for achieving radical cure of HPC under the premise of preserving organ function [4,5]. However, tumor cells tend to resist to X-ray radiation during treatment, ultimately leading to tumor recurrence or metastasis [6]. Protective autophagy serves as a critical mechanism responsible for radiotherapy resistance by maintaining cellular homeostasis and tumor cell survival under hostile conditions [7,8]. Inhibiting protective autophagy in tumor cells represents a crucial strategy for enhancing radiosensitivity and improving survival rates in HPC.

Autophagy inhibitors have been successfully used to suppress protective autophagy in tumor cells, thus enhancing the radiotherapy efficacy [[9], [10], [11]]. However, due to the lack of tumor-targeted delivery, autophagy inhibitors accumulate in normal tissues such as the brain and heart, thereby causing severe side effects. To address this challenge, researchers have developed stimuli-responsive nanocarriers with controlled release capabilities [[12], [13], [14]]. Although these nanocarriers can realize responsive release of autophagy inhibitors, they are dependent on either exogenous or endogenous stimuli alone and the selectivity for tumor cells is yet to be improved. Moreover, it is critical to note that the release process requires a relatively long reaction time, due to either slow reaction kinetics or the involvement of multiple steps of reaction. In fact, the initiation of radiotherapy triggers protective autophagy in tumor cells rapidly, accompanied by the upregulation and activation of relevant pro-survival factors and signaling pathways [15]. Therefore, the precise and rapid release of autophagy inhibitors in tumors is crucial for achieving radiosensitization in radiotherapy.

**Scheme 1:**
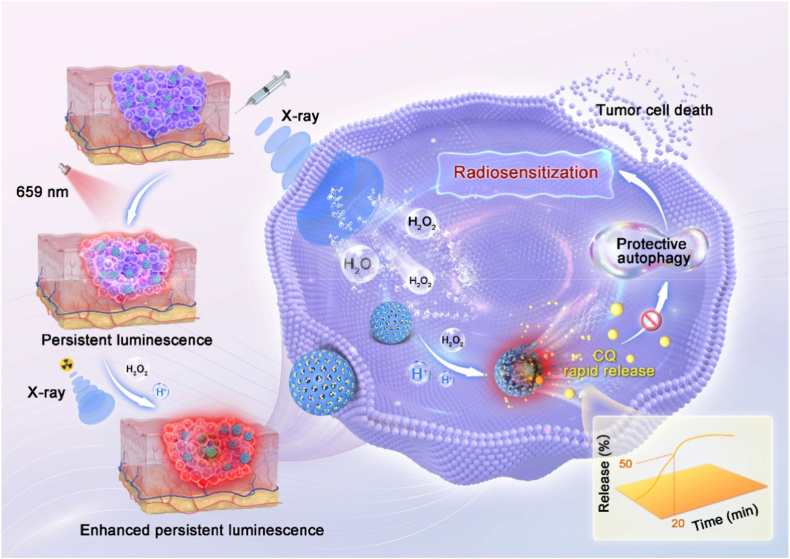
Schematic diagram of the precise and rapid release of autophagy inhibitors by ZMC under external X-ray irradiation and the internally slightly acidic TME.

Polymers with polysulfide bonds are widely utilized in tumor microenvironment (TME)-responsive nanocarriers owing to their susceptibility to oxidation by H_2_O_2_ or reduction by glutathione (GSH) [[16], [17], [18], [19]]. Because of the low endogenous concentrations of H_2_O_2_ or limited reducing capacity of GSH, the relatively slow reaction kinetics lead to drug release profiles exceeding 6 h [20,21], accompanied by low release efficiency. During radiotherapy, ionizing radiation generates substantial H_2_O_2_ clusters [22], while the acidic condition enhances the oxidation capability of H_2_O_2_ [23,24]. We speculate that polysulfide bonds-containing polymers will undergo rapid redox reactions under intense X-ray radiation in acidic TME. In addition, precise imaging guidance serves as a critical method for improving release accuracy [[25], [26], [27]]. Based on this, we develop a multifunctional afterglow nanocarrier loaded with autophagy inhibitors. The guidance of precise imaging and activation of therapeutic X-ray initiate the precise and rapid release of the autophagy inhibitors (Scheme 1). The nanocarrier was constructed using near-infrared persistent luminescence nanoparticles Zn_1.1_Ga_1.8_Ge_0.1_O_4_:0.5%Cr (ZGGC) as the imaging probe, which was coated with tetrasulfide-incorporated organic-inorganic hybrid silica to form ZGGC@MON (ZM). The autophagy inhibitor chloroquine (CQ) was subsequently loaded into the mesoporous silica to form ZGGC@MON-CQ (ZMC). Under precise X-ray irradiation, a substantial amount of H_2_O_2_ clusters is generated in tumor cells and the acidic TME further accelerates the oxidation of tetrasulfide bonds by H_2_O_2_. The silica layer degrades specifically in response to high concentrations of H_2_O_2_ and the mildly acidic environment, endowing ZMC with high imaging signal-to-noise ratio of 50.7 and achieving CQ release efficiency of 50% within 20 min. It is demonstrated that ZMC significantly enhanced radiotherapy efficacy while reducing the required radiation dose in vivo. This work develops a high-performance nanocarrier responsive to both endogenous and exogenous stimuli, which enables effective radiosensitization. This work also offers a novel strategy for the construction of precise and rapid release drug delivery systems for treatment of tumors.

## 2 Materials and methods

**Chemicals.** Zinc nitrate hexahydrate (Zn(NO_3_)_2_•6H_2_O, 99.99%), gallium nitrate hydrate (Ga(NO_3_)_3_•xH_2_O, 99.99%), germanium oxide (GeO_2_, 99.99%), chromium(Ⅲ) nitrate nonahydrate (Cr(NO_3_)_3_•x9H_2_O, 99.99%), sodium hydroxide (NaOH, 99.9%), and ammonia solution (GR, 25–28%) and cetyltrimethylammonium bromide (CTAB) were acquired from Aladdin. Tetraethyl orthosilicate (TEOS), triethanolaminse (TEA) and chloroquine (CQ) were acquired from Sigma-Aldrich. Bis[3-(triethoxysilyl)propyl] tetrasulfide (BTES) was purchased from Macklin. Phosphate-buffered solution (PBS, 10 × 0.01 M, pH:7.2–7.4) was purchased from Solarbio. The precursor solution of 0.4 mol L^−1^ Na_2_GeO_3_ was prepared by dissolving GeO_2_ powder in 2 mol L^−1^ NaOH solution. 30% hydrogen peroxide (H_2_O_2_) was obtained from Sinopharm Chemical Reagent Co., Ltd (Beijing, China). CYTO-ID autophagy detection kit 2.0 was acquired from Enzo Life Sciences Inc (Farminsgdale, NY, USA). 4, 6-diaminso-2-phenylindole (DAPI), radio immunoprecipitation assay (RIPA) buffer, bicinchoninic acid (BCA) protein assay kit, enhance chemiluminescence (ECL) kit and hydrogen peroxide (H_2_O_2_) Assay Kit were obtained from Beyotime Biotechnology. Calcein AM-PI dye was obtained from Thermo Fisher Scientific. Cell Counting kit-8 (CCK-8) and all cell culture supplies were obtained from ZetaLife. 3-Methyladenine (3-MA) was purchased from KKL Med Inc. All chemicals were used directly as received without further purification. Deionized water (resistivity∼18.2 MΩ) was used for total experiments.

**Instruments.** Transmission electron microscopy (TEM) images were taken by a Hitachi H-7650 (Hitachi Co., Ltd., Japan). X-ray powder diffraction (XRD) patterns were detected by a minsiflex 600 X-ray diffractometer (Rigaku Co., Ltd., Japan). The pore-size distributions and N_2_ adsorption–desorption isotherms dates were measured by Autosorb-IQ (Quantachrome Instruments, USA). UV-vis absorption spectra absorption spectra were obtained from Cary 5000 spectrophotometer (Agilent, USA). The afterglow spectrums were measured by Edinburgh FS5 fluorescence spectrophotometer (Edinburgh Instruments Co., Ltd., UK). Zeta potential and hydrodynamic diameter of nanoparticles were evaluated using dynamic laser scattering (Brookhaven, USA). The cell images were collected on a Confocal laser scanning microscopy (CLSM) (Nikon, Japan). The X-ray was conducted on a minsi-X X-ray tube (Amptek, Inc., USA). Western blot assays were carried out using an HC (high current) power supply cell set (BioRad, USA). Quantitative analyses of elements were determinsed by ICP-OES (Horiba Jobin Yvon S.A.S, France). Energy-dispersive spectroscopy (EDS) elemental mapping and High-angle annular dark-field scanning transmission electron microscopy (HAADF-STEM) were collected by an FEI Talos F200s transmission electron microscope (Thermo Fisher, USA). Fourier-transform infrared (FTIR) spectra were detected by thermo Nicolet iS50 FT-IR spectrometer (Thermo Fisher Co., Ltd., USA).

**Cell culture and Animals.** FaDu cells were purchased from the Wuhan Pricella Biotechnology Co.,Ltd. Cell culture reagents were purchased from Gibco, USA. All cells were cultured as monolayers in Minimum Essential Medium (MEM) supplemented with 10% fetal bovine serum (FBS) at 37 °C with 5% CO_2_. SPF KM mice (6 weeks, 20-22 g) and male nude mice (6 weeks, 20-22 g) were purchased from Wu's for Experimental Animals. All animal experiments were performed strictly in accordance with the protocols of the National Regulations on the Care and Use of Laboratory Animals. All the animal experiments were approved by the Animal Ethics Committee of the Second Affiliated Hospital of Fujian Medical University (20240615).

### 2.1 Synthesis of Zn_1.1_Ga_1.8_Ge_0.1_O_4_: 0.5%Cr persistent luminescent nanoparticles (ZGGC-PLNPs)

The precursor mixture was prepared by sequentially introducing 2.2 mL of Zn(NO_3_)_2_ solution (1 mol L^−1^), 7.2 mL of Ga(NO_3_)_3_ solution (0.5 mol L^−1^), 0.5 mL of Na_2_GeO_3_ solution, and 0.5 mL of Cr(NO_3_)_3_ solution (20 mmol L^−1^) into a beaker containing 49.6 mL of deionized water under continuous stirring. Subsequently, ammonia solution was rapidly injected under vigorous stirring to adjust the pH of the mixed solution to approximately 6.5. After continuous stirring at room temperature for 2 h, the mixture was transferred into a Teflon-lined stainless-steel autoclave. The hydrothermal reaction was conducted at 200 °C for 12 h, followed by natural cooling to room temperature. The as-synthesized ZGGC-PLNPs were harvested by centrifugation (10,000 rpm, 10 min) and subsequently purified three times with deionized water. The final product was collected after centrifugation at 10,000 rpm for 10 min and then dried into powder in an oven at 60 °C.

### 2.2 Synthesis of ZGGC@MON

50 mg of the as-obtained ZGGC powder and 0.75 g of CTAB were accurately weighed and dispersed in 30 mL of ultrapure water and then sonicated for 30 min. Subsequently, 0.375 mL of TEA (25 % w/v) was added, and the mixture was stirred in an oil bath at 60 °C and 300 rpm for 1 h. Dissolve 600 μL of TEOS and 600 μL of BTES in 8 mL of cyclohexane, then slowly add this mixture drop by drop to the reaction system and stir for 24 h. Finally, the sample ZGGC@MON was collected by centrifugation at 10,000 rpm for 10 min. The obtained samples were alternately washed with water and ethanol more than three times. To remove the template of CTAB from the mesoporous pores, the sample was dispersed in a 0.6% NH_4_NO_3_/ethanol solution, stirred in an oil bath at 60 °C for 2 h, and then washed alternately with water and ethanol for three times. Re-disperse the obtained samples in the 0.6% NH_4_NO_3_/ethanol solution and repeat the above operation twice.

### 2.3 Loading of CQ

0.5 mL of CQ solution (20 mg mL^−1^) was added to ZGGC@MON (1 mg mL^−1^) solution, and the mixture was stirred at room temperature in the dark for 24 h. Subsequently, the obtained ZGGC@MON-CQ was centrifuged at 10,000 rpm for 10 min and washed for three times with deionized water to remove the excess CQ. The washing supernatants were collected to calculate the drug loading ratio. Finally, the precipitate was collected and dried for further experiments.

### 2.4 Cytotoxicity of ZGGC@MON

FaDu cells were seeded into 96-well plates at a density of 5 × 10^3^ cells per well and cultured for 24 h under standard culture conditions (37 °C, 5% CO_2_). Subsequently, replace with fresh medium containing ZGGC@MON (0, 12.5, 25, 50, 100, 200 μg mL^−1^). After 24 h, the supernatant was replaced with fresh medium containing 10% CCK-8 for 2 h at 37 °C, and the absorbance at 450 nm was measured using a microplate reader. All measurements were performed three times, and the viability was expressed as a percentage relative to untreated cells.

### 2.5 The treatment process of ZGGC@MON degradation

H_2_O_2_-induced degradation of ZGGC@MON: 3 mg mL^−1^ ZGGC@MON was dispersed in 500 μM H_2_O_2_ solution and subjected to ultrasonic treatment for 10 min to ensure thorough mixing and reaction. Subsequently, the mixture was centrifuged at 10,000 rpm for 10 min to separate the supernatant. The precipitate was collected and dried under vacuum at 60 °C for 2 h. To investigate the degradation behavior of ZGGC@MON, X-ray photoelectron spectroscopy (XPS) was used to analyze the chemical valence states of sulfur elements in the precipitate, and the occurrence of the degradation process was confirmed through the valence state change. In addition, the release of silicon element in the supernatant after degradation was determined by inductively coupled plasma optical emission spectrometry (ICP-OES).

### 2.6 Release of CQ

1 mL of ZGGC@MON-CQ with a concentration of 2 mg mL^−1^ was added to 1 mL of PBS (pH = 6.5) containing H_2_O_2_ (100 μM) and mixed thoroughly. The reaction was terminated after 1, 10, 30, 60, 120 and 240 min, respectively. After centrifugation at 10000 rpm for 10 min, the supernatant was collected, appropriately diluted with PBS, and the absorbance at 344 nm was measured using a UV-vis spectrophotometer to calculate the release rate.

### 2.7 Intracellular antitumor experiment

Cell viability was assessed by the standard CCK-8 assay. FaDu cells were seeded in 12-well plates (1 × 10^5^ cells per well) and incubated for 24 h in a humidified condition (37 °C, 5% CO_2_). After adhesion, the cells were randomly divided into 6 groups: (1) control, (2) CQ, (3) ZGGC@MON-CQ, (4) X-ray, (5) ZGGC@MON + X-ray, and (6) ZGGC@MON-CQ + X-ray. The doses of ZGGC@MON-CQ, ZGGC@MON, and CQ were 200 μg mL^−1^, 200 μg mL^−1^, and 25 μg mL^−1^, respectively. Cells were treated with ZGGC@MON-CQ, ZGGC@MON and CQ for 6 h and then irradiated with X-ray at a cumulative dose of 3 Gy. After incubation for 24 h, the medium was replaced with fresh medium containing 10% (v/v) CCK-8 reagent and incubated for another 2 h at 37 °C. Finally, the absorbance at 450 nm was measured using a microplate reader. All experiments were conducted three times, and cell viability was expressed as a percentage relative to the untreated group.

### 2.8 Live/dead cell staining

FaDu cells were seeded in 12-well plates (1 × 10^5^ cells per well) and cultured for 24 h (37 °C, 5% CO_2_) to ensure complete adherence. Subsequently, the cells were randomly divided into six groups: (1) control, (2) CQ, (3) ZGGC@MON-CQ, (4) X-ray, (5) ZGGC@MON + X-ray, and (6) ZGGC@MON-CQ + X-ray. Different nanomaterials at 200 μg mL^−1^ and CQ at 25 μg mL^−1^ were added respectively, and X-ray irradiation of 3 Gy was carried out after an interval of 6 h. After incubation for 24 h, live cell/dead cell staining was performed using a working solution of 2 μM Calcein-AM and 4 μM ethidium homodimer-1 (EthD-1). Live cells (green fluorescence; Ex/Em = 495/515 nm) and dead cells (red fluorescence; Ex/Em = 495/635 nm) were discriminated by fluorescence microscopy and quantified via ImageJ software.

### 2.9 Cyto-ID autophagy detection

FaDu cells were seeded in 12-well plates (1 × 10^5^ cells per well) and cultured for 24 h (37 °C, 5% CO_2_). After adhesion, the cells were incubated with ZGGC@MON (200 μg mL^−1^) for 30 min, followed by irradiation with a single 3 Gy dose of X-ray. After incubating the cells for 0, 10, 30, 60, 120, and 180 min respectively, the culture medium was replaced with fresh medium containing 2 μM CYTO-ID® Green Detection Reagent and 1 μM Hoechst 33342 nuclear stain. After 30 min of incubation at 37 °C, the cells were gently washed three times with PBS and fixed with 4% paraformaldehyde for 15 min at room temperature. After an additional nuclear counterstain with DAPI (1 μg mL^−1^, 5 min), autophagic flux was evaluated by CLSM. Green fluorescent puncta (CYTO-ID) were quantified using ImageJ to assess the level of autophagy.

### 2.10 Determination of H_2_O_2_

To determine the relationship between the intracellular H_2_O_2_ generation under radiotherapy and the radiation dose, FaDu cells were seeded in 6-well plates at a density of 2 × 10^6^ cells per well and cultured for 24 h (37 °C, 5% CO_2_). The cells were then randomly divided into three groups: (1) control, (2) X-ray (5 min), and (3) X-ray (10 min). Six hours after irradiation, the cells were harvested by trypsinization, transferred into 1.5 mL microcentrifuge tubes, and centrifuged at 300×*g* for 5 min. The supernatant was discarded, and the cells was lysed by adding 100-200 μL of H_2_O_2_ assay lysis buffer per 1 × 10^6^ cells, followed by thorough homogenization on ice to ensure complete cell disruption. The lysate was subsequently centrifuged at 10,000 rpm for 5 min at 4 °C, and the supernatant was transferred to a quartz cuvette. Absorbance at 450 nm was immediately recorded using a microplate reader to determine H_2_O_2_ concentration.

### 2.11 pH response characteristics

To further investigate the pH-responsive release characteristics of CQ from ZGGC@MON-CQ, 2 mg mL^−1^ of ZGGC@MON-CQ was added to PBS solutions containing 500 μM H_2_O_2_ with different pH values (pH = 3.5, 6.5, 7, 9.5, 11.5), followed by thorough mixing and 5-min incubation at room temperature. Finally, the supernatant was collected by centrifugation at 10,000 rpm for 10 min, diluted, and the absorbance at 344 nm was measured under a UV spectrophotometer. The release of CQ under different pH values was calculated. To simultaneously monitor the pH-dependent degradation of the mesoporous organosilica framework, 3 mg mL^−1^ ZGGC@MON was dispersed in PBS solutions (pH = 3.5, 6.5, 7.0, 9.5, 11.5) containing 500 μM H_2_O_2_ and incubated at 37 °C. At predetermined intervals (1, 10, 30, 60, 120, and 240 min), samples were centrifuged at 10,000 rpm for 10 min and the supernatants were collected. The cumulative concentration of Si in the supernatants was determined by ICP-OES to assess the dissolution kinetics of the nanocarrier across the pH range.

### 2.12 Western blotting

The treated cells were harvested, washed twice with ice-cold PBS, and lysed on ice for 10 min using radio-immunoprecipitation assay (RIPA) buffer supplemented with protease and phosphatase inhibitors. Cell suspensions were then sonicated for 1 min (30 % amplitude, pulse-on 2 s, pulse-off 3 s) to ensure complete lysis, followed by centrifugation at 12,000 rpm for 15 min at 4 °C. The supernatants were mixed with 5 × Laemmli loading buffer, boiled at 100 °C for 5 min. The proteins were separated by electrophoresis on a 15% sodium dodecyl sulfate (SDS)-polyacrylamide gel and subsequently transferred onto a polyvinylidene fluoride (PVDF) membrane. Following transfer, the membrane was incubated with a primary antibody at 4 °C for 12 h, followed by incubation with a secondary antibody at 25 °C for 1 h. After washing five times with TBST (each time for 5 min), membranes were incubated with HRP-conjugated secondary antibodies (1:10000) for 1 h at 25 °C, washed again (5 × 5 min), and visualized using an enhanced chemiluminescence (ECL) detection kit on a ChemiDoc™ MP Imaging System.

### 2.13 Animal experiments

Animal experiments were conducted in accordance with the guidelines of the Chinese National Regulations on the Care and Use of Laboratory Animals and were approved by the Animal Ethics Committee of the Affiliated Hospital of Fujian Medical University (20240615). SPF KM mice (6 weeks, 20–22 g) and male nude mice (6 weeks, 20–22 g) were obtained from Wu's for Experimental Animals.

To establish a subcutaneous tumor model of hypopharyngeal carcinoma in nude mice, 1 × 10^7^ FaDu cells suspended in 100 μL of sterile PBS were subcutaneously inoculated into the right axilla of male BALB/c nude mice (6–8 weeks, 18–20 g). Tumor growth was monitored by digital calipers every two days. When the tumor volume reached approximately 100 mm^3^ (length × width^2^ × 0.5), the tumor model was successfully established. To evaluate the enhanced afterglow performance of ZGGC@MON in a real tumor environment, 50 μl of PBS containing ZGGC@MON (500 μg) was subcutaneously injected into the tumor site and non-tumor area of the same tumor-bearing nude mouse, respectively. The mice were then anesthetized with 2 % isoflurane gas at given time intervals, illuminated with 659 nm LED for 3 min, and then exposed for 180 s using an IVIS Lumina II imaging system to acquire persistent luminescence images.

For the in vivo therapeutic study, tumor-bearing nude mice were randomly divided into 7 groups (n = 6 per group): (1) control, (2) PBS (+), (3) ZGGC@MON, (4) ZGGC@MON-CQ, (5) ZGGC@MON-CQ (+), (6) ZGGC@MON (+), and (7) CQ (+). The nanoparticles were administrated intratumoral at the dose of 20 mg kg^−1^, the CQ in group (7) was delivered via tail vein injection at a dosage of 25 mg kg^−1^, and the local irradiation (6 Gy per time, total 18 Gy) with an X-ray tube were indicated as "(+)". The specific treatment protocols for each group are detailed in Table S2. Treatments were repeated every 3 days for a total of three fractions. Body weight and tumor dimensions were recorded every other day and the tumor volume was calculated as V = (length × width^2^)/2. 72 h after the final treatment, mice were euthanized. The heart, spleen, liver, lung, kidney, and tumor tissues of mice were harvested, fixed in 4 % paraformaldehyde for 24 h, paraffin-embedded, sectioned at 4 μm, and stained with hematoxylin and eosin (HE) for histopathological analysis.

To further evaluate whether ZGGC@MON-CQ-mediated radiosensitization could enable reduction of X-ray dosage in tumor therapy, tumor-bearing nude mice were randomly allocated into three groups (n = 6 per group): (1) control, (2) PBS + 18 Gy, and (3) ZGGC@MON-CQ + 12 Gy. ZGGC@MON-CQ was administered at 20 mg kg^−1^ every three days, followed by X-ray irradiation (6 Gy per fraction) 6 h later. Group 2 received three fractions of X-ray irradiation to achieve 18 Gy, whereas group 3 received only two fractions to reach 12 Gy. Body weight and tumor dimensions were measured every other day, with tumor volume calculated as V = (length × width^2^)/2.

## 3 Results and discussion

### 3.1 Construction of the multifunctional nano-drug delivery system of ZMC

**Fig. 1:**
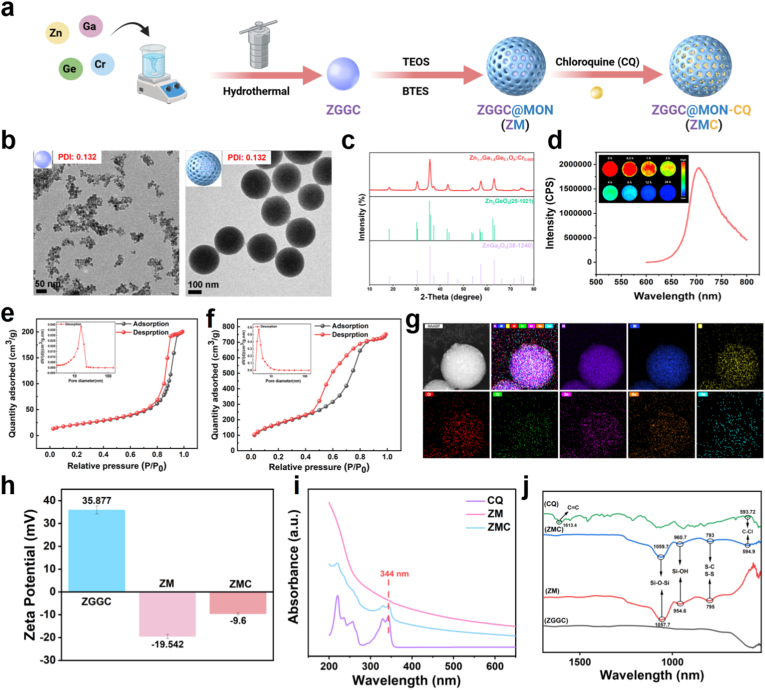
Synthesis of ZMC and its characterization. (**a**) Schematic illustration of the preparation process of ZMC. (**b**) TEM images and dispersion characterization of ZGGC and ZM. (**c**) XRD pattern of ZGGC. (**d**) Afterglow emission spectrum of ZGGC (excitation at 659 nm) and persistent luminescence images at different time points after 5 min of excitation at 659 nm (exposure time: 60 s). (**e**) N_2_ adsorption-desorption isotherms of ZGGC and (**f**) ZM (the inset shows the corresponding pore size distribution). (**g**) EDS elemental mapping images of ZMC. (**h**) Zeta potential analysis of ZGGC, ZM, and ZMC (mean ± SD, n = 3, ∗∗∗*p* < 0.001). (**i**) UV-vis absorption spectra of CQ, ZM and ZMC solutions. (**j**) FTIR spectroscopic analysis of ZGGC, ZM, ZMC, and CQ.

Persistent luminescence nanoprobes achieve high signal-to-noise ratio imaging by overcoming the interference of background fluorescence, making them the outstanding novel materials in the field of bioimaging [28]. In this work, near-infrared persistent luminescence nanomaterial of ZGGC was synthesized via a hydrothermal method. A mesoporous organosilica network (MON) was subsequently coated onto its surface using a two-phase method to form ZM. Finally, chloroquine (CQ) was loaded into the mesopores to construct the multifunctional nanocarrier ZMC (Fig. 1a). The synthesized ZGGC exhibited uniform spherical morphology with a hydrodynamic diameter of 154.2 ± 0.8 nm (Fig. 1b, Fig. S1). The X-ray diffraction (XRD) measurements confirmed that ZGGC possesses a typical cubic spinel structure (JCPDS file No. 25-1021 and 38-1240), further verifying its successful synthesis (Fig. 1c). After coating with MON, the surface of ZGGC formed mesopores capable of loading small molecules, while significantly improving its dispersibility. The transmission electron microscopy (TEM) and dynamic light scattering (DLS) results revealed that ZM exhibits a spherical morphology with uniform size and homogeneous dispersion, demonstrating an average hydrodynamic diameter of 346.5 ± 4.4 nm (Fig. 1b, Fig. S2). Notably, compared to the polydispersity index (PDI) of ZGGC (0.312 ± 0.015), the PDI of ZM significantly decreased to 0.097 ± 0.004, indicating that the coating of MON remarkably improved both the stability and dispersibility of ZM. After 5 min of excitation at 659 nm, ZGGC exhibited a strong afterglow emission peak at 698 nm (Fig. 1d), demonstrating its significant advantage for operation within the biological window that the absorption and scattering by biological tissues are minimized. Afterglow decay images collected by a CCD camera over 24 h (inset, Fig. 1d) revealed that ZGGC maintained a strong and stable afterglow emission for up to 30 min after the excitation light was turned off. In addition, the afterglow emission intensity could be recovered upon re-excitation. These results demonstrate that ZGGC exhibits excellent near-infrared afterglow emission properties, supporting its potential for guiding drug release and enabling in vivo imaging monitoring. Elemental mapping images and energy dispersive spectroscopy (EDS) analysis confirmed the homogeneous distribution of the elements of Zn, Ga, Ge, Si, Cr, S, and O within ZM, further verifying the successful coating of the MON layer (Fig. S3a and b). Notably, the metal components are Zn, Ga, Ge and Cr from the luminescence center of ZGGC. The atomic numbers of Zn, Ga, Ge, and Cr are 30, 31, 32, and 24, respectively, all of which are below 40. Compton scattering dominates for Zn, Ga, Ge, and Cr, with the photoelectric effect contributing minimally. Consequently, effective localized radiation dose enhancement is hardly to achieve [29]. To further investigate the changes in surface structure, the specific surface area, pore volume, and pore size of ZGGC and ZM were measured respectively. Nitrogen adsorption-desorption isotherms (Fig. 1e and f) confirmed the successful formation of the mesoporous structures in ZM after MON coating. Based on Brunauer-Emmett-Teller (BET) and density functional theory (DFT) methods, the specific surface area was determined to be 940.072 m^2^ g^−1^ and the pore volume and pore size of ZM were calculated to be 1.273 cm^3^ g^−1^ and 4.319 nm (Supplementary Table S1) respectively, further indicating that ZM show well-defined mesoporous structures that are suitable for drug loading.

Achieving high-efficiency drug loading is critical for the successful construction of a nano-drug delivery platform [30]. In this work, CQ was loaded into the mesopores of ZM to form ZMC by electrostatic adsorption. The average hydrodynamic diameter of ZMC increased to 385.9 ± 4.5 nm compared to that of ZM (Fig. S4). EDS elemental mapping confirmed the homogeneous distribution of S and Cl elements on the surface of ZM (Fig. 1g). Furthermore, the zeta potentials of ZGGC and ZM were measured to be +35.877 mV and −19.542 mV (Fig. 1h). After immersing ZM in the CQ solution, the zeta potential of the particles shifted to −9.6 mV. Additionally, a distinct absorption peak emerged at approximately 344 nm for ZM after CQ loading (Fig. 1i), which is attributed to the characteristic absorption of CQ [12]. A new absorption peak also appeared at 594.9 nm in the fourier transform infrared spectroscopy (FTIR) spectrum, corresponding to the C–Cl stretching vibration [31] (Fig. 1j). These results collectively confirm the successful loading of CQ and the successful preparation of the multifunctional nanocarrier of ZMC.

### 3.2 ZMC exhibits a stimuli-responsive release property specifically triggered by **abundant** H_2_O_2_ generated under X-ray irradiation and the mildly acidic TME

**Fig. 2:**
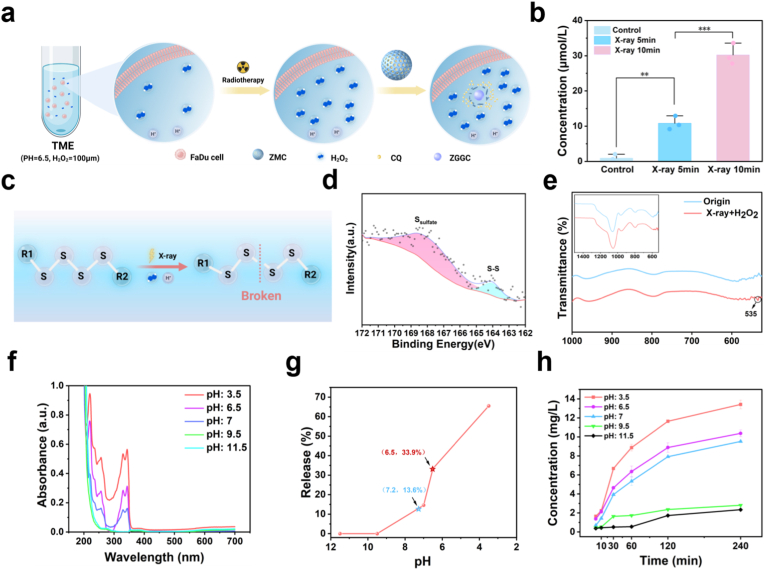
The dual responsive properties of ZMC to both internal and external stimuli under X-ray irradiation. (**a**) Schematic diagram illustrating the increase in H_2_O_2_ concentration in the TME and the degradation of the organosilicon layer of ZMC after X-ray irradiation. (**b**) The variation of H_2_O_2_ levels under different X-ray irradiation times (mean ± SD, n = 3; ∗∗*p* < 0.01, ∗∗∗*p* < 0.001). (**c**) Schematic illustration of the tetrasulfide bond cleavage in the organosilicon layer of ZMC under X-ray excitation in the condition of TME. (**d**) XPS spectra of ZMC after reacted with 500 μM H_2_O_2_. (**e**) FTIR spectra of ZMC and the solid products obtained after its reaction with 500 μM H_2_O_2_. (**f**) UV-vis absorption spectra of CQ released from ZMC at different pH values (H_2_O_2_: 500 μM). (**g**) Graph of the relationship between the CQ release rate of ZMC and pH value (H_2_O_2_: 500 μM). (**h**) The variation curves of Si content in ZMC degradation products with time at different pH values (H_2_O_2_: 500 μM; mean ± SD; n = 3).

Sulfur-containing polymers are frequently utilized in studies concerning GSH-responsive release systems; however, their release rates are generally slow, often requiring more than 6 h to achieve effective release [32]. Our previous research demonstrated that MON is able to effectively undergo a redox reaction with H_2_O_2_ [33]. Although the concentrations of H^+^ and H_2_O_2_ in the TME are significantly higher than those in normal tissues, the level of H_2_O_2_ remains insufficient to degrade MON. Besides the suppression of GSH, promoting the generation of a large amount of H_2_O_2_ also plays an important role in enhancing tumor therapy [34,35]. It is noteworthy that the therapeutic X-ray irradiation positively contributes to the formation of reactive oxygen species (ROS) that predominated by H_2_O_2_ [22,36]. Therefore, we suppose that under external irradiation of X-ray, higher concentrations of H_2_O_2_ clusters generated within the TME, leading to the degradation of the outer layer of ZMC and subsequent release of CQ loaded in the mesopores (Fig. 2a). To validate the hypothesis, we applied external X-ray irradiation under simulated TME conditions. The results demonstrated a significant increase in H_2_O_2_ concentration (∗∗*p* < 0.01), which was positively correlated with the irradiation time (∗∗∗*p* < 0.001) (Fig. 2b). We propose that the responsiveness of ZMC to H_2_O_2_ attributes to the relatively low bond energies of both S–S and S–C bonds in MON (each below 300 kJ mol^−1^), which allow high concentrations of H_2_O_2_ to cleave the tetrasulfide bonds, ultimately leading to the disruption of MON (Fig. 2c). To validate this hypothesis, X-ray photoelectron spectroscopy (XPS) analysis on the solid products after the complete reaction of ZMC with H_2_O_2_ was conducted. As shown in Fig. 2d, in addition to the S–S peak at 163.5 eV, a distinct peak attributed to sulfur oxides emerged at 168.7 eV, indicating the oxidation of tetrasulfide bonds [37]. The result confirms that high concentrations of H_2_O_2_ disrupt the tetrasulfide bonds in MON and further oxidize them to higher-valence sulfur compounds, providing strong support for our hypothesis. FTIR spectrum further proved the cleavage of tetrasulfide bonds, with a characteristic peak at 535 nm assigned to the bending vibration of S–O bonds [38] (Fig. 2e). The above data confirm the degradation mechanism of ZMC: X-ray irradiation significantly increases the level of H_2_O_2_ in the tumor microenvironment. The tetrasulfide bonds doped in the ZMC shell undergo redox reactions with the highly oxidizing H_2_O_2_, thereby breaking the bonds and causing degradation. The collapse of the ZMC outer shell releases the loaded autophagy inhibitor.

Interestingly, the H_2_O_2_-responsive release of CQ from ZMC exhibits pH dependence. As the concentration of H^+^ increased, the amount of CQ released following the reaction of ZMC with H_2_O_2_ gradually increased (Fig. 2f). At the physiological pH of normal tissues (7.2), the release rate of CQ was 13.6%. In contrast, under the acidic TME (pH 6.5), the release rate significantly increased to 33.9% (Fig. 2g), further enhancing the tumor-specific release specificity of CQ. The variation curve of the content of Si element in ZMC degradation products over time, measured by ICP-OES under different pH conditions (Fig. 2h), similarly demonstrated that the reaction between ZMC and H_2_O_2_ intensifies with increasing acidity. This pH-dependent properties can be explained by the underlying oxidative mechanism of H_2_O_2_. During the reduction reaction of H_2_O_2_ (Eq. (1)), the Nernst equation for the reaction is given by Eq. (2), where $\varphi_{\;H2O2\;/H2O}^{\theta }$ is 1.776 V, and the value of $\varphi_{H2O2\;/H2O}$ is positively correlated with the concentration of H^+^. Therefore, an increase in H^+^ concentration enhances the oxidative capacity of H_2_O_2_, which accounts for the acid-responsive release behavior of CQ from ZMC.

(1) $$H_{2}O_{2}+2H^{+}+2e^{‐}\;\to \;2H_{2}O$$

(2) $$\varphi_{H2O2\;/H2O}=\varphi_{H2O2\;/H2O}^{\theta }+\frac{0.059}{2}\;{\mathrm{lg}\left[H^{+}\right]}^{2}$$

In summary, a multifunctional nanocarrier of ZMC with excellent imaging performance, capable of being excited by X-rays, and responsive to both H_2_O_2_ and H^+^ has been successfully prepared. ZMC simultaneously achieves dual response to external therapeutic radiation and internal TME, showing great potential for achieving precise and rapid release of autophagy inhibitors.

### 3.3 The stimuli-responsive release of CQ from ZMC is precise and rapid

**Fig. 3:**
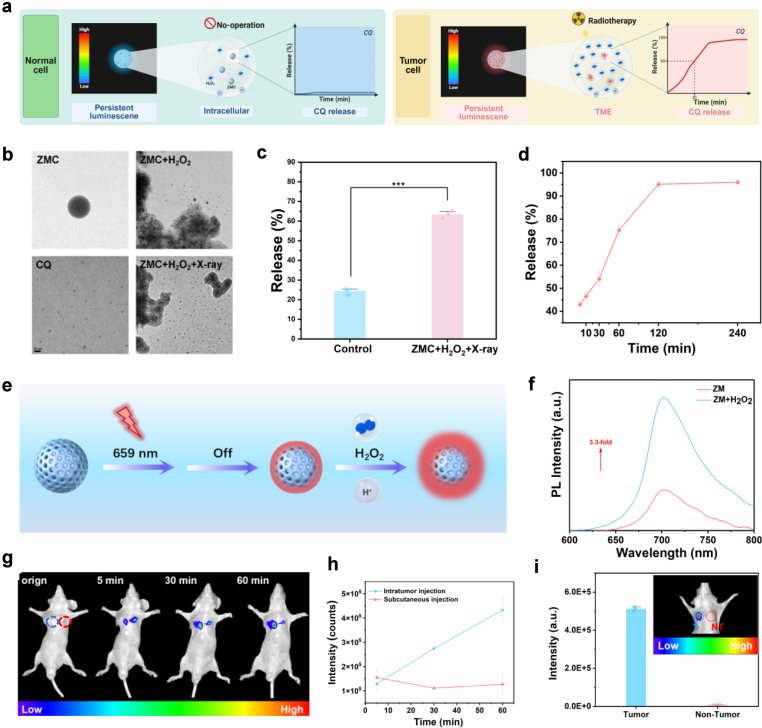
ZMC enables rapid and precise release of CQ upon X-ray activation. (**a**) Schematic illustration of the differences in imaging and CQ release of ZMC in tumors and normal tissues. (**b**) TEM images showing CQ release from ZMC in response to H_2_O_2_ (500 μM) with or without X-ray irradiation. (**c**) CQ release profiles of ZMC in PBS and a radiotherapy-simulated TME (H_2_O_2_: 500 μM) after 60 min of treatment (mean ± SD, n = 3; ∗∗∗*p* < 0.001). (**d**) Time-dependent CQ release kinetics of ZMC in the radiotherapy-simulated TME (H_2_O_2_: 500 μM) (mean ± SD, n = 3). (**e**) Schematic diagram of ZM exhibiting afterglow emission upon cessation the excitation at 659 nm and its responsive luminescence enhancement to H_2_O_2_ within the acidic TME. (**f**) Afterglow emission spectra of ZM in the absence or presence of H_2_O_2_ (500 μM) (excitation at 659 nm). (**g**) Afterglow images of mouse following intratumoral (the blue circle) and subcutaneous (the red circle) injections of ZM (excitation at 659 nm, exposure time: 60 s, dose: 20 mg kg^−1^). (**h**) The variation curve of afterglow intensity calculated based on the images in Fig. 3g (mean ± SD, n = 3). (**i**) Comparison of afterglow emission intensities between tumor tissue and normal tissue after ZMC administration (inset: CCD camera image of mouse afterglow, blue circle: tumor tissue, red circle: normal tissue) (excitation at 659 nm, exposure time: 60 s, dose: 20 mg kg^−1^). (For interpretation of the references to colour in this figure legend, the reader is referred to the Web version of this article.)

Drug carriers with intelligent response release functions have extensive applications in the field of precise delivery [[39], [40], [41], [42]]. Drug delivery platforms that integrate both imaging and multi-stimuli-responsive capabilities significantly enhance the precision of drug release and therapeutic efficacy [43]. Owing to the exceptional imaging capability and intelligent stimulus-responsive property of ZMC, we hypothesize that the afterglow emission intensity is markedly enhanced in tumor tissues compared to normal tissues. Furthermore, ZMC demonstrates a dual-responsive capability to both external X-ray irradiation and the internal TME, enabling precise and rapid release of the loaded CQ (Fig. 3a). To verify the hypothesis, ZMC was incubated in a PBS solution simulating the TME (H_2_O_2_ = 500 μM, pH = 6.5), and the TEM imaging revealed the collapse of the outer silica layer structure of ZMC (Fig. 3b). Meanwhile, dispersed particulate releases were observed around, the morphology and contrast of which were consistent with that of CQ. We therefore conclude that these released particles were CQ that encapsulated in ZMC. The use of external X-ray irradiation significantly enhanced the observed phenomena: the collapse of the silica layer was more thorough, and the release amount of CQ was markedly increased compared to treated with H_2_O_2_ alone. To quantitatively investigate the CQ release profile of ZMC in the TME versus normal tissue conditions, X-ray irradiation was applied under simulated TME. After 1 h of incubation, the CQ release rate in the TME reached 63.33%, demonstrating a statistically significant difference (∗∗∗*p* < 0.001) compared to that in a normal PBS solution environment (Fig. 3c). The enhanced release is attributed to the high concentration of H_2_O_2_ generated in the TME during radiotherapy, which triggers the cleavage of tetrasulfide bonds, leading to the structural collapse of the silica layer (Fig. 2a–e) and substantial release of the encapsulated CQ consequently. These findings further confirmed that ZMC utilizes external X-ray irradiation and the internal mildly acidic TME as intelligent responsive triggers, enabling precise and controllable release of CQ in tumor.

The release kinetics of CQ during treatment are critical for achieving effective radiosensitization. To evaluate the release rate of CQ, ZMC was incubated in a PBS solution simulating the TME during radiotherapy (H_2_O_2_: 500 μM). The supernatant was collected at various time intervals and analyzed by UV-vis absorption spectroscopy. The results demonstrated that approximately 50% of CQ was released within 20 min, and the release rate gradually approached 100% after 4 h (Fig. 3d). These findings indicate that ZMC exhibits rapid stimuli-responsive release characteristics and efficiently releases CQ, which attribute to the high concentrations of H_2_O_2_ accelerate the cleavage rate of tetrasulfide bonds, highlighting its unique advantages as a promising nanocarrier for future tumor therapy.

As a persistent luminescence nanoparticle capable of overcoming background fluorescence interference to achieve high signal-to-noise ratio imaging, ZM exhibits persistent afterglow emission upon cessation of excitation at 659 nm. Owing to the bond-cleavage characteristics of ZM under X-ray irradiation in the TME, we propose that the afterglow emission of ZM will be further enhanced (Fig. 3e). To proof this hypothesis, ZM nanoparticles were dispersed in PBS (pH = 7) and H_2_O_2_ solution (500 μM, pH = 6.5), respectively. It is found that upon the addition of H_2_O_2_, the afterglow emission intensity of the ZM solution increased by 3.3-fold (Fig. 3f), demonstrating a significant enhancement of the luminescence in the TME. Furthermore, the afterglow emission intensity of ZGGC was significantly higher than that of ZM (Supplementary Fig. S5), suggesting that the silica coating exerts shielding and absorption effects on the afterglow luminescence [33]. Therefore, the mechanism underlying the enhanced luminescence of ZM in the TME was attributed to the blocking and absorption of afterglow by the silica layer. Upon oxidation and cleavage of the tetrasulfide bonds by H_2_O_2_, the silica layer collapses, thereby exposing the luminescent centers and resulting in enhanced emission. To further investigate the imaging sensitivity of ZM in solid tumors, equal doses (20 mg kg^−1^) of the sample were administered via subcutaneous and intratumoral injections in mice. As shown in Fig. 3g, in vivo imaging analysis clearly revealed a gradual increasement of the afterglow signal in the intratumoral region over time, whereas the signal at the subcutaneous injection site remained unchanged. Quantitative analysis further revealed the time-dependent trend of afterglow signal intensity (Fig. 3h), confirming the high imaging sensitivity of ZM at the tumor site. Ultimately, the detected tumor-to-normal tissue (T/NT) signal ratio reached a remarkably high value of 50.7 (Fig. 3i). This value significantly surpasses the threshold established by the Rose criterion [44,45], ensuring high-contrast and precise tumor imaging.

These data collectively demonstrate that the developed autophagy inhibitor carrier of ZMC exhibits highly efficient responsiveness to both X-ray activation and the mildly acidic TME, enabling high-sensitivity imaging as well as rapid and precise release of the autophagy inhibitor.

### 3.4 The released CQ effectively inhibits the protective autophagy induced by the nanocarriers under radiotherapy

**Fig. 4:**
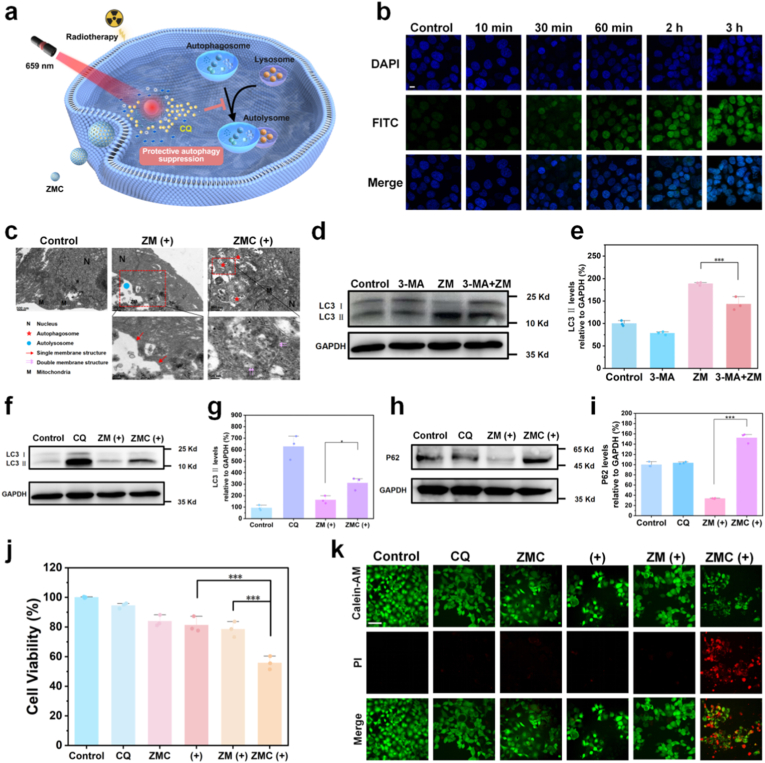
CQ released from ZMC effectively inhibits the nanocarrier-induced protective autophagy under radiotherapy. (**a**) Schematic illustration of the process of ZMC releases CQ to suppress protective autophagy, under the guidance of persistent luminescence imaging and X-ray excitation. (**b**) Confocal fluorescence images of autophagosomes generated in FaDu cells treated with PBS (Control) or ZM (200 μg mL^−1^) combined with radiotherapy (6 Gy) (scale bar: 10 μm). (**c**) TEM images of FaDu cells treated with PBS (Control), ZM with radiotherapy, or ZMC with radiotherapy for 24 h (“+”: radiotherapy of 6 Gy). (**d**) Western blot analysis of LC3 and GAPDH in FaDu cells with different treatments (CQ: 30 μM; ZM: 200 μg mL^−1^; ZMC: 200 μg mL^−1^; “+”: radiotherapy of 6 Gy). (**e**) Quantitative analysis of LC3-Ⅱ levels based on Fig. 4d (mean ± SD, n = 3; ∗*p* < 0.05). (**f**) Western blot analysis of LC3 and GAPDH in FaDu cells with different treatments (3-MA: 5 mM; ZM: 200 μg mL^−1^). (**g**) Quantitative analysis of LC3-Ⅱ levels based on Fig. 4f (mean ± SD, n = 3; ∗∗∗*p* < 0.001). (**h**) Western blot analysis of p62 and GAPDH in FaDu cells with different treatments (CQ: 30 μM; ZM: 200 μg mL^−1^; ZMC: 200 μg mL^−1^; “+”: radiotherapy of 6 Gy). (**i**) Quantitative analysis of p62 levels based on Fig. 4h (mean ± SD, n = 3; ∗∗∗*p* < 0.001). (**j**) Cell viability of FaDu cells with different treatments (CQ: 30 μM; ZM: 200 μg mL^−1^; ZMC: 200 μg mL^−1^; “+”: radiotherapy of 6 Gy). (**k**) Confocal fluorescence images of FaDu cells stained with Calcein AM (live cells, green fluorescence, Ex/Em = 495/515 nm) and Propidium Iodide (dead cells, red fluorescence, Ex/Em = 495/635 nm) after different treatments (“+”: radiotherapy of 6 Gy; scale bar: 50 μm). (For interpretation of the references to colour in this figure legend, the reader is referred to the Web version of this article.)

Autophagy serves as an essential cell self-protection mechanism and plays a crucial defense role in response to external stimuli [46]. Protective autophagy in tumor cells promotes resistance to radiotherapy [9]. This work proposes a novel radiosensitization strategy: utilizing persistent luminescence nanoparticles loaded with CQ to achieve precise tumor imaging under external excitation at 659 nm. Upon precise activation by X-rays and in synergy with internal modulation of TME, the rapid release of CQ is triggered, effectively inhibiting the protective autophagy process and thereby significantly enhancing radiosensitivity (Fig. 4a). To demonstrate the significance of rapid release of the autophagy inhibitor, we investigated the kinetics of autophagy induction. The results indicated that a significant increase in autophagosome number could be observed as early as 30 min after cells co-treated with the ZM and radiotherapy (Fig. 4b). Quantitative analysis further confirmed a significant enhancement in the fluorescence intensity of autophagosome at 30 min (∗∗∗*p* < 0.001) (Fig. S6), with the autophagy level gradually increasing over time. The induction of protective autophagy simultaneously activates pro-survival signaling pathways. Consequently, the rapid release of the autophagy inhibitor is crucial for achieving effective radiosensitization.

To illustrate the cellular uptake mechanism of ZMC, co-staining experiment upon lysosome and FITC-modified ZMC were performed. As shown in Fig. S7, when cells were co-incubated with FITC-modified ZMC, the intracellular uptake of ZMC increased over time and reached the maximum at 6 h. The co-localization results with lysosomes showed that ZMC and lysosomes were consistently located, indicating that ZMC entered the lysosomes after being internalized by the cells. This also reveals the successful fusion of lysosomes with autophagosomes containing ZMC. Ultrastructural analysis of tumor cells (Fig. 4c) visually demonstrates the process of autophagy. In PBS treated cells, structures associated with autophagy were rarely observed, and the cells maintained intact organelle architectures. In contrast, cells treated with ZM combined with radiotherapy exhibited typical single-membraned autolysosomes, within which the nanomaterial ZM was visible, indicating enhanced autophagic activity and successful fusion of autophagosomes with lysosomes. Conversely, in the group of cells co-treated with ZMC and radiotherapy, accumulation of double-membraned autophagosomes was observed. The phenomenon can be attributed to the effective inhibition of autophagosome-lysosome fusion by CQ released from ZMC, thereby blocking autophagic flux.

To further explore the mechanism of autophagy process regulated by CQ, the dynamic progression of autophagic flux was investigated. During autophagosome formation, LC3-Ⅰ in the cytoplasm is converted to membrane-bound LC3-Ⅱ, and autophagosomes ultimately fuse with lysosomes for degradation [47,48]. Firstly, cells were co-treated with ZM and an inhibitor 3-MA that suppresses the conversion of LC3-Ⅰ to LC3-Ⅱ. As shown in Fig. 4d, the ZM treated group exhibited a significant increase in LC3-Ⅱ levels, indicating that ZM induces elevated autophagy level. Co-treatment with 3-MA markedly reduced ZM-induced LC3-Ⅱ accumulation (∗∗∗*p* < 0.001) (Fig. 4e), confirming that the increase in LC3-Ⅱ level resulted from the enhancement of upstream autophagosome formation. Subsequently, we explored the downstream process of autophagosome-lysosome fusion. We found that ZMC loaded with the autophagy inhibitor effectively suppressed the increase in LC3-Ⅱ levels induced by ZM and radiotherapy (∗*p* < 0.05) (Fig. 4f and g). This effect is attributed to CQ-mediated alkalinization of lysosomes and inhibition of autophagic degradation, leading to intracellular accumulation of LC3-Ⅱ and blockade of autophagic flux [49,50]. Additionally, we assessed the degradation of the autophagy substrate p62. As demonstrated in Fig. 4h, the co-treated group of ZM and radiotherapy showed reduced expression of p62, whereas loading with CQ significantly promoted p62 accumulation (∗∗∗*p* < 0.001) (Fig. 4i). Collectively, these analyses indicate that ZM induces a complete autophagy process during radiotherapy, while CQ released from ZMC under external X-ray irradiation and internal TME conditions effectively inhibits the degradation of autophagosome, thereby blocking autophagic flux.

Autophagy induced by external stimuli may promote cell death or enhance cell survival [15,51]. Therefore, before modulating autophagy, it is essential to clarify the type of autophagy induced by ZM under radiotherapy. We first evaluated the cytotoxic effects of ZM. Due to the nanomaterials are primarily metabolized in the liver [52],the impact of ZM on the viability of both FaDu cells and normal hepatocytes (AML-12) were investigated. The results demonstrated that the cell viability remains above 98% even at a relative high concentration of 200 μg mL^−1^, indicating excellent biocompatibility of ZM (Fig. S8). Subsequently, the effects of ZMC introduction on cell viability relative to radiotherapy alone was compared. The results revealed that co-treatment with ZMC significantly enhanced radiosensitivity, reducing cell viability from 80% to 55% (Fig. 4j). Additionally, ZMC significantly improved the therapeutic efficacy of the carrier ZM under radiotherapy, strongly demonstrating that ZM induces protective autophagy under the irradiation of X-ray. Moreover, CQ released from ZMC effectively inhibit the protective autophagy, thereby conferring a radiosensitizing effect. Fluorescence images of live/dead staining visually confirmed that CQ released from ZMC could suppress protective autophagy during the treatment, effectively enhancing radiotherapy efficiency (Fig. 4k). These findings confirm that ZMC significantly enhances radiotherapy efficacy by releasing CQ to inhibit the protective autophagy triggered by ZM and radiotherapy, holding substantial potential for reversing radiotherapy resistance in cancer therapy.

### 3.5 ZMC significantly enhances the radiosensitivity of tumors in vivo, thereby reducing the required radiation dosage during radiotherapy

**Fig. 5:**
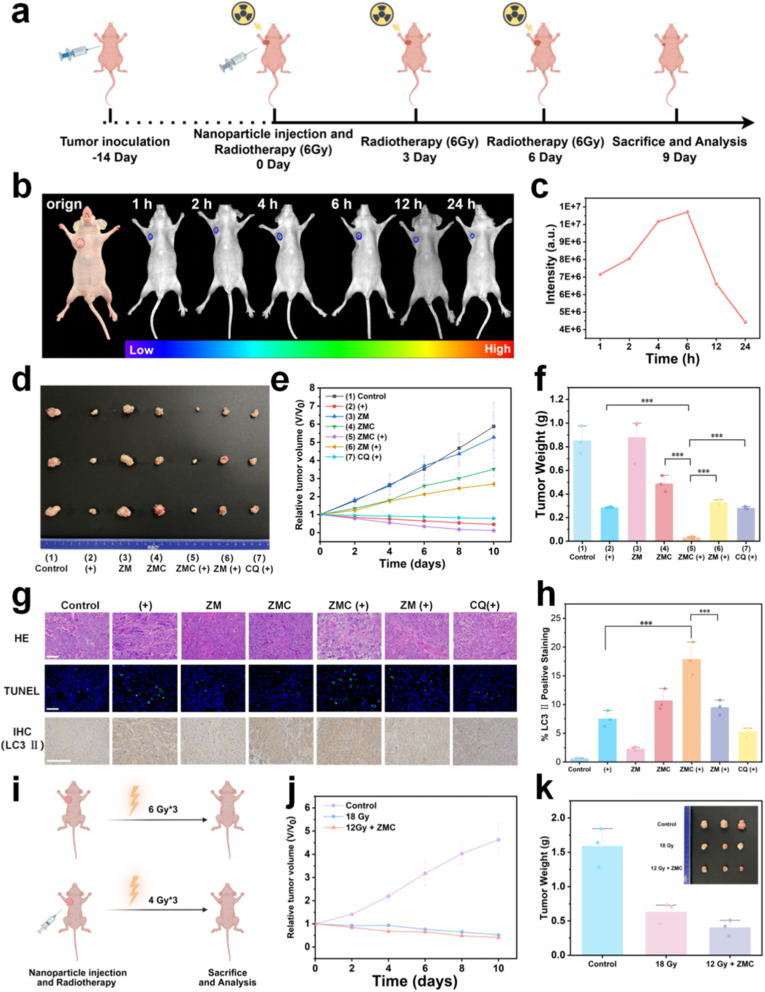
The carrier ZMC effectively radiosensitized HPC in vivo and reduced the radiation dose. (**a**) Establishment of the FaDu tumor model and the tumor treatment protocol. (**b**) Afterglow imaging of tumors in nude mice at different time points after injection of ZM (excitation at 659 nm) (ZM: 20 mg kg^−1^). (**c**) Quantitative analysis based on the afterglow intensity in Fig. 5b. (**d**) Representative images of tumors dissected from nude mice on day 9. (**e**) Variation of relative tumor volume over time during the treatment period (mean ± SD, n = 3) (CQ: 25 mg kg^−1^, ZM: 20 mg kg^−1^, ZMC: 20 mg kg^−1^, total radiation dose: 18 Gy, 6 Gy every 3 days, “+”: radiotherapy). (**f**) Tumor weight in different groups of nude mice on day 9 (mean ± SD, n = 3, ∗∗∗*p* < 0.001) (“+”: radiotherapy). (**g**) Histological staining of tumors from different groups: HE (scale bar: 100 μm), TUNEL (scale bar: 100 μm), and IHC (LC3-II) (scale bar: 200 μm), (“+”: radiotherapy). (**h**) Quantitative analysis of LC3-Ⅱ based on IHC staining results in Fig. 5g (“+”: radiotherapy) (mean ± SD, n = 3, ∗∗∗*p* < 0.001). (**i**) Schematic diagram of the grouping and the corresponding treatment protocol for tumor-bearing nude mice. (**j**) Variation of relative tumor volume over time during the treatment period (mean ± SD, n = 3) (ZMC: 20 mg kg^−1^). (**k**) Tumor images (inset) and weight (mean ± SD, n = 3) from different groups of nude mice on day 9.

To further validate the antitumor efficacy of ZMC combined with radiotherapy in vivo, we subsequently evaluated the synergistic antitumor effects of ZMC and radiotherapy in a tumor-bearing nude mouse model. The designed animal model and therapeutic protocol are illustrated in Fig. 5a. The mice were randomly divided into seven groups (n = 6): (1) control; (2) PBS + X-ray; (3) ZM; (4) ZMC; (5) ZMC + X-ray; (6) ZM + X-ray; (7) CQ + X-ray. Three sessions of radiotherapy (6 Gy each time) were respectively administered after the injection of ZM or ZMC. The treatment protocols for each group are detailed in Table S2. Precise imaging of the tumor in vivo before treatment is a prerequisite for guiding therapy [53,54]. To determine the optimal time point of imaging, 50 μL of ZM (20 mg kg^−1^) was injected into the tumors. Optical imaging of the tumors was performed at different time points after excitation at 659 nm for 5 min. As shown in Fig. 5b and c, the intensity of optical signal in the tumor gradually increased, reaching the maximum intensity at 6 h. Therefore, 6 h is identified as the optimal window for subsequent combination therapy with radiotherapy. The increasing intensity before 6 h is attributed to the cleavage of the outer organosilicon layer of ZM in the TME, leading to enhanced intensity of afterglow emission.

Fig. 5d shows representative images of tumors after the imaging-guided therapy. A significant reduction of tumor volume was observed in Group (5), indicating that ZMC significantly enhanced the antitumor efficacy of radiotherapy. The variations of tumor volume during the treatment are shown in Fig. 5e. The tumor suppression efficiency in Group (5) was significantly higher than that in Groups (4) and (6), demonstrating that both X-ray activation and CQ loading are necessary conditions for ZM to achieve optimal therapeutic efficacy. Notably, even when CQ was administered intravenously in combination with radiotherapy (Group (7)), the antitumor effect remained inferior to that of Group (5). The result indicates the precision and efficiency of CQ release from ZMC. The optimal antitumor performance of Group (5) is attributed to the rapid and precise release of CQ from ZMC triggered by X-ray, which effectively inhibits protective autophagy, thereby serving as a radiosensitization strategy. Furthermore, the statistical analysis of tumor weights provides additional support for the conclusion (Fig. 5f). To further validate these findings, tumor tissues were analyzed using HE and TUNEL staining. The results revealed that the ZMC + X-ray group exhibited significantly higher levels of necrosis of tumor cell. To elucidate the mechanism underlying the enhanced radiotherapy efficacy, immunohistochemical (IHC) analysis of LC3-Ⅱ in tumor tissues was performed. As demonstrated in Fig. 5g, the ZMC and X-ray co-treated group showed a significantly higher accumulation of LC3-Ⅱ compared to the ZM and X-ray co-treated group, which is attributed to the released CQ from ZMC inhibiting the fusion of autophagosomes and lysosomes, thereby blocking the degradation of LC3-Ⅱ and effectively suppressing the protective autophagy. Quantitative analysis of LC3-Ⅱ levels further confirmed these conclusions (Fig. 5h).

To further verify whether ZMC-mediated radiosensitization could reduce the required radiation dose and mitigate associated side effects, tumor-bearing nude mice were randomly divided into three groups (n = 6): (1) control; (2) 18 Gy; (3) ZMC + 12 Gy. The therapeutic efficacy between the high-dose group (18 Gy) and the ZMC-treated low-dose group (ZMC + 12 Gy) was specifically investigated (Fig. 5i). As shown in Fig. 5j, the variation trends of tumor volume during the treatment period were similar between groups (2) and (3). The picture (inset, Fig. 5k) of excised tumors after treatment revealed the sizes of the low-dose group (3) was even smaller than the high-dose group (2). It is demonstrated that in combination with ZMC administration, only two-thirds of the radiation dose (12 Gy) is required to achieve an antitumor efficacy comparable to that of the full dose (18 Gy). The above results indicate that the significant radiosensitizing effect of ZMC, achieving low-dose radiotherapy and reducing radiation side effects.

### 3.6 ZM and ZMC demonstrate excellent biocompatibility

**Fig. 6:**
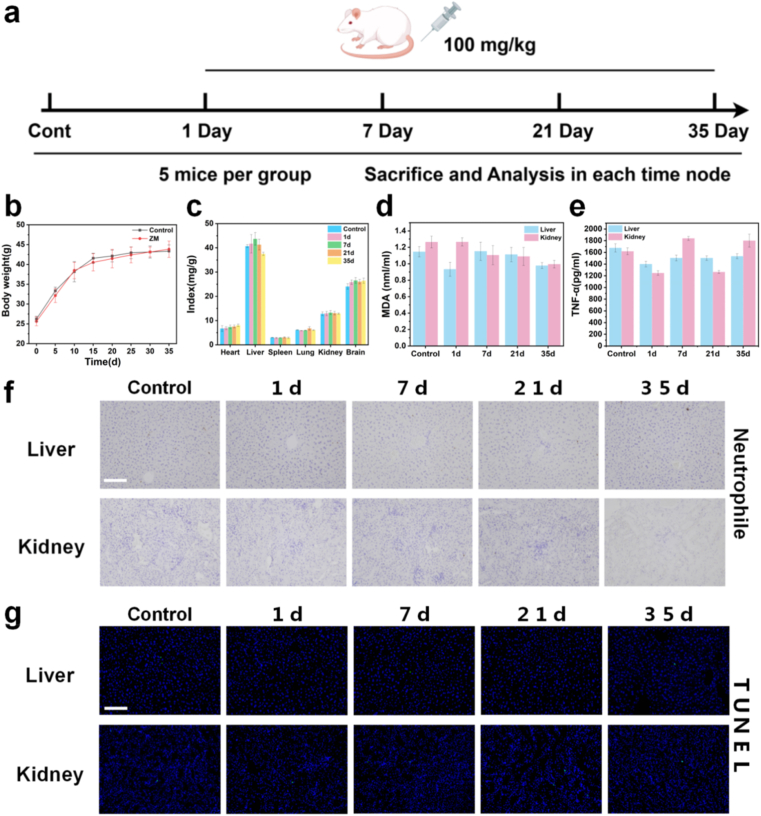
ZM demonstrates excellent biocompatibility. (**a**) Schematic diagram of the experimental design for in vivo biosafety assessment of ZM. (ZM: 100 mg kg^−1^). Body weight changes of mice over 35 days (**b**) and organ coefficients at various time points (**c**) (mean ± SD, n = 3) (ZM: 100 mg kg^−1^). Contents of MDA (**d**) and TNF-α (**e**) in liver and kidney tissues measured by ELISA. (**f**) Immunohistochemical evaluation of neutrophil infiltration in the liver and kidney using the LY6G marker (scale bar: 100 μm). (**g**) Cell apoptosis in liver and kidney tissues (TUNEL assay) (scale bar: 100 μm).

Prior to clinical application, a comprehensive evaluation of the biosafety of nanodrug delivery platform is inevitable. To assess the sub-chronic toxicity of ZM, KM mice were randomly divided into five groups (n = 5) and administered 100 mg kg^−1^ ZM via tail vein injection. Tissues were collected and analyzed on days 1, 7, 21, and 35 post-injections (Fig. 6a). The results showed that body weight and organ coefficients remained relatively stable, indicating normal overall health of the mice (Fig. 6b and c). Histopathological examination, hematological analysis, and biochemical profiling demonstrated neglectable acute or chronic toxicopathological changes in major organs (heart, liver, spleen, lungs, kidneys, and brain) at any time point (Fig. S9). Furthermore, all hematological parameters fluctuated within normal ranges (Fig. S10). Serum biochemical markers reflected liver and kidney function also remained within normal limits (Fig. S11a–d). These results collectively indicate that ZM exhibits good biocompatibility in vivo. The liver and kidney, as the main organs for metabolism and biotransformation, are vulnerable to the influence of exogenous substances [55,56]. We further evaluated the safety of liver and kidney by analyzing oxidative stress, inflammatory responses, and apoptosis. Enzyme-linked immunosorbent assay (ELISA) results showed that malondialdehyde (MDA) and tumor necrosis factor (TNF-α) levels in liver and kidney tissues did not fluctuate significantly within 35 days after ZM administration (Fig. 6d and e), suggesting ZM does not cause obvious oxidative stress or inflammatory reactions. Immunohistochemical stain revealed that the number of LY6G-positive cells was comparable to that in the control group (Fig. 6f). TUNEL apoptosis assays showed no significant apoptotic signals in ZM-treated liver or kidney tissues at any time point (Fig. 6g). These findings demonstrate that ZM, exhibits negligible toxicity and satisfactory biosafety at the therapeutic dose, supporting its potential for further biomedical applications.

In fact, the biosafety of ZMC was also evaluated during the treatment of tumor-bearing mouse. It is proved that the body weight of nude mice in all groups administered with ZMC exhibit normal level (Fig. S12). In addition, the organ coefficients remained within the normal range, suggesting minimal treatment-related side effects (Fig. S13). Following co-treatment with ZMC and radiotherapy, histopathological analysis of major organs revealed neglect lesions, indicating that ZMC possesses favorable biocompatibility during tumor therapy (Fig. S14). The above data confirm that the carrier ZM and the nanomedicine ZMC exhibit excellent biological safety, representing a novel nanodrug with potential for clinical application.

Based on the findings presented above, this work demonstrates that ZMC serves as a safe and efficient radiosensitizer, which significantly enhances radiotherapeutic efficacy in an animal model of HPC. The underlying mechanism is attributed to its ability to precisely block the autophagic flux in tumor cells, thereby reversing the protective autophagy response under radiation. The combined strategy effectively reduces the required radiation dose, consequently mitigating side effects, and provides a solid foundation for developing novel cancer therapeutic approaches based on the rapid and precise regulation of autophagy.

ZMC as a multifunctional nanocarrier for the controlled release of autophagy inhibitors, holds broad application prospects in clinical tumor therapy. Notably, autophagy is closely associated with the immunosuppressive environment [57,58]. Autophagy inhibition can reverse or weaken the immunosuppressive microenvironment established by tumors. Therefore, the multifunctional nanocarrier of ZMC has great application prospects in sensitizing immuno-radiotherapy. In the future research, we will focus on developing the potential applications of ZMC in various tumor treatment regimens, particularly in immuno-radiotherapy.

## 4 Conclusions

In summary, to address the issues of non-specific release and slow release kinetics (exceeding 6 h) of autophagy inhibitors, we developed a multifunctional near-infrared persistent luminescence nanocarrier ZMC which demonstrated dual response to external X-ray radiation source and internal acidic TME. Under the irradiation of X-ray, a substantial number of H_2_O_2_ clusters were generated locally in tumor cells. The acidic TME further enhanced the oxidation reactivity of the H_2_O_2_ clusters, leading to rapid cleavage of the outer organosilica layer of ZMC. Simultaneously, the intensity of afterglow is also enhanced at the tumor site, achieving high signal-to-noise ratio (SNR = 50.7) imaging of the tumor. CQ loaded in mesoporous silica is also released rapidly (achieving 50% release within 20 min), efficiently inhibiting protective autophagy and thereby enhancing radiosensitivity. ZMC demonstrate highly effective radiosensitization in HPC, enabling a reduction in the required radiation dose. In addition, the subchronic toxicity evaluation suggested the excellent biocompatibility of ZMC. This work provides a novel strategy for constructing drug carriers capable of rapid and precise release.

## CRediT authorship contribution statement

**Xinyuan Luo:** Conceptualization, Data curation, Formal analysis, Funding acquisition, Investigation, Methodology, Project administration, Resources, Software, Supervision, Validation, Visualization, Writing – original draft, Writing – review & editing. **Rui Rong:** Conceptualization, Data curation, Formal analysis, Funding acquisition, Investigation, Methodology, Project administration, Resources, Software, Supervision, Validation, Visualization, Writing – original draft, Writing – review & editing. **Jiao Zhou:** Conceptualization, Formal analysis, Investigation. **Qiongling Huang:** Conceptualization, Formal analysis, Investigation. **Yanjun Huang:** Conceptualization, Formal analysis, Investigation. **Xiaofang Chen:** Conceptualization, Formal analysis, Investigation. **Ming Lu:** Conceptualization, Formal analysis, Investigation. **Shenjiong Ruan:** Conceptualization, Formal analysis, Investigation. **Kehui Chen:** Conceptualization, Formal analysis, Investigation. **Yuanchang Zhou:** Conceptualization, Formal analysis, Investigation. **Zexin Li:** Conceptualization, Formal analysis, Investigation. **Jiwang Zhang:** Conceptualization, Formal analysis, Investigation. **Yun Zhang:** Resources, Supervision, Writing – review & editing. **Chaohui Zheng:** Resources, Supervision, Writing – review & editing.

## Declaration of competing interest

The authors declare that they have no known competing financial interests or personal relationships that could have appeared to influence the work reported in this paper.

## Data Availability

Data will be made available on request.
