# Supplementary material for: Precise and rapid release of X-ray activated autophagy inhibitors for radiotherapy sensitization of hypopharyngeal carcinoma
Source: Mater Today Bio. 2026 Mar 25;38:103070. doi: 10.1016/j.mtbio.2026.103070 (PMC13068793; doi:10.1016/j.mtbio.2026.103070)
Supplement: Multimedia component 1 [file mmc1.docx]

Revised Supporting Information

**Precise and rapid release of X-ray activated autophagy inhibitors for** **radiotherapy sensitization of hypopharyngeal carcinoma**

Xinyuan Luo ^1, #^, Rui Rong ^2, 3, 4, #, *^, Jiao Zhou ^1^, Qiongling Huang ^1^, Yanjun Huang ^1^, Xiaofang Chen ^1^, Ming Lu ^1^, Shenjiong Ruan ^1^, Kehui Chen ^1^, Yuanchang Zhou ^1^, Zexin li ^1^, Jiwang Zhang ^1^, Yun Zhang ^2, 3, *^, Chaohui Zheng ^1, *^

^1^ Department of Otolaryngology, the Second Affiliated Hospital, Fujian Medical University, Quanzhou, Fujian 362000, China

^2^ State Key Laboratory of Structural Chemistry, Fujian Institute of Research on the Structure of Matter, Chinese Academy of Sciences, Fuzhou, Fujian 350002, China

^3^ Xiamen Key Laboratory of Rare Earth Photoelectric Functional Materials, Xiamen Institute of Rare Earth Materials, Xiamen, Fujian 361021, China

^4^ First Affiliated Hospital of Xiamen University, School of Medicine, Xiamen University, Xiamen 361003, P.R. China

^*^E-mail: zchfydfsey@163.com; rongrui@mail.ustc.edu.cn; zhangy@fjirsm.ac.cn

#: The authors contributed equally: Xinyuan Luo and Rui Rong

**Supporting Figures and Table**

**
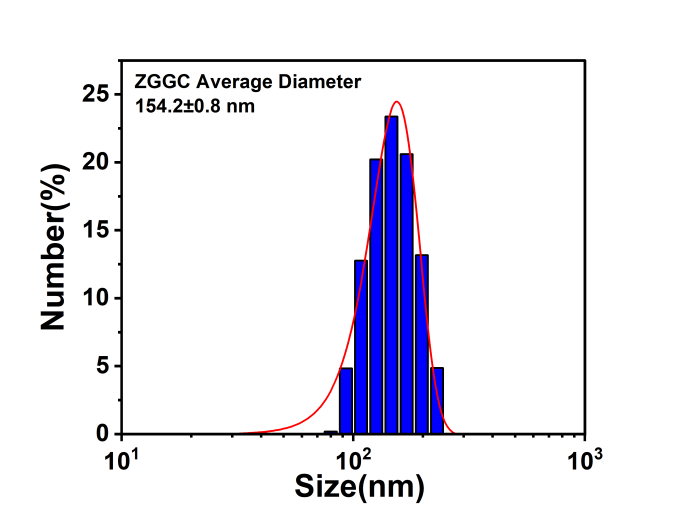
**

**Fig. S1.** DLS analysis of ZGGC.​


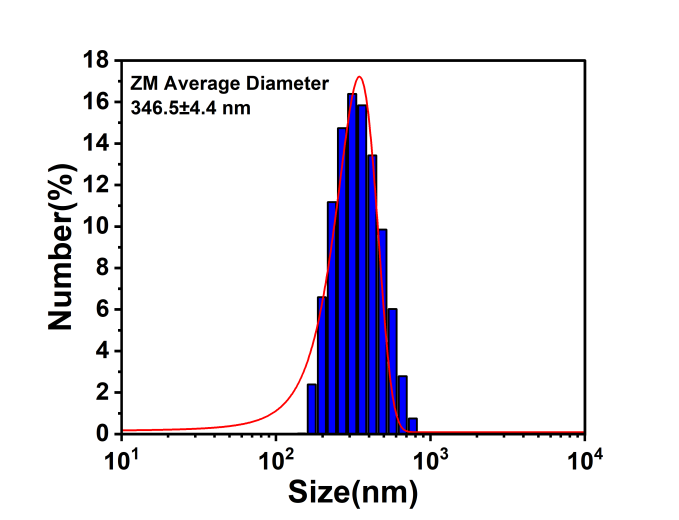


**Fig. S2.** DLS analysis of ZM.​

**
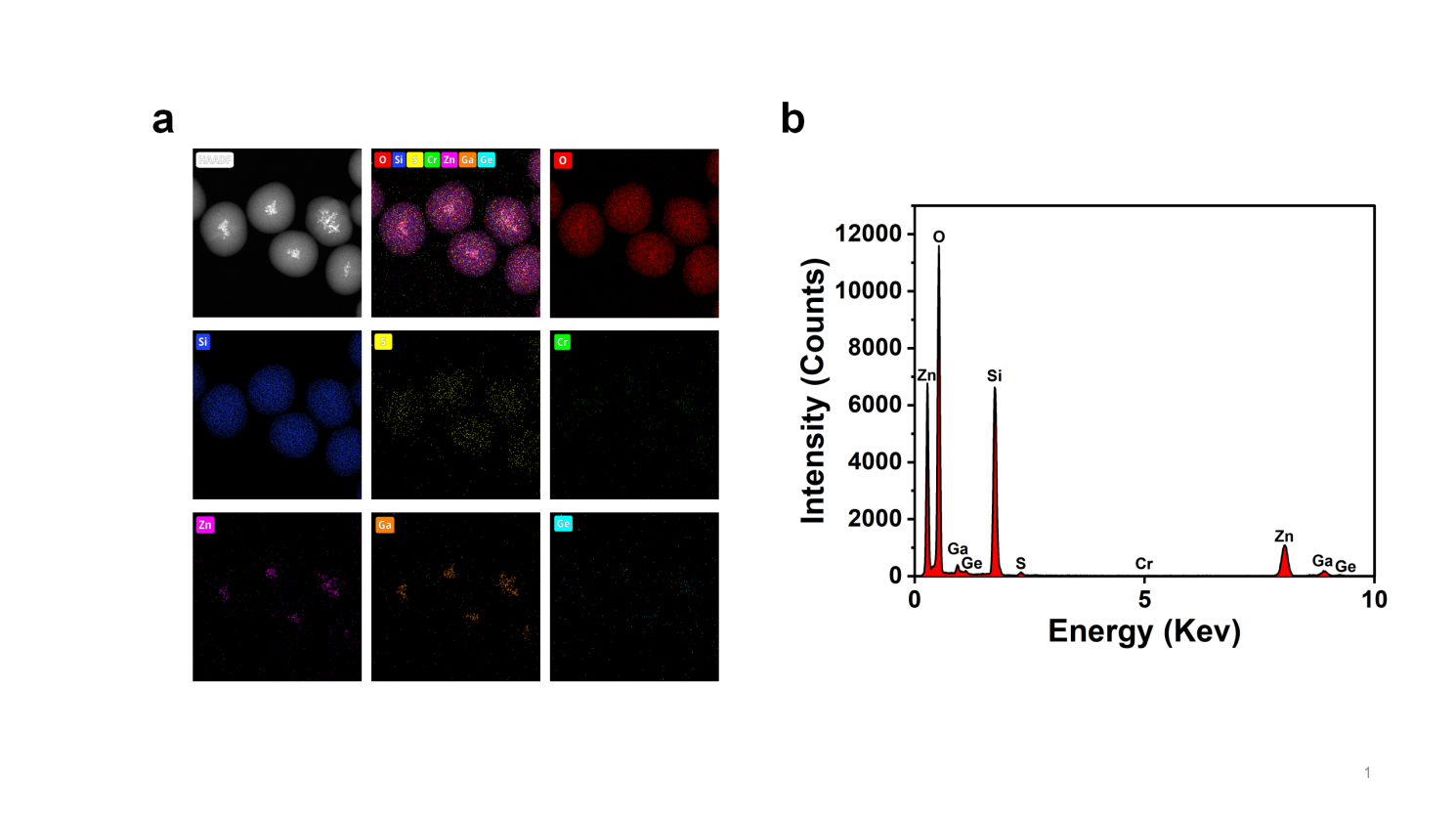
**

**Fig. S3.** (a) EDS elemental mapping images of ZM and (b) HAADF-STEM spectrum analysis.​

**
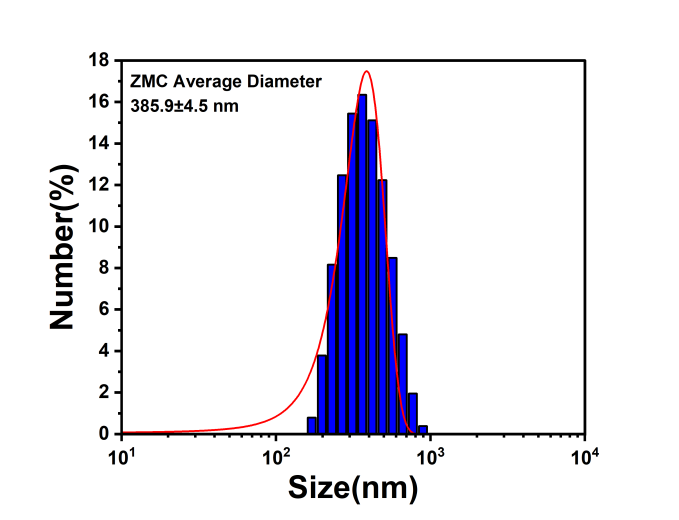
**

**Fig. S4.** DLS analysis of ZMC.


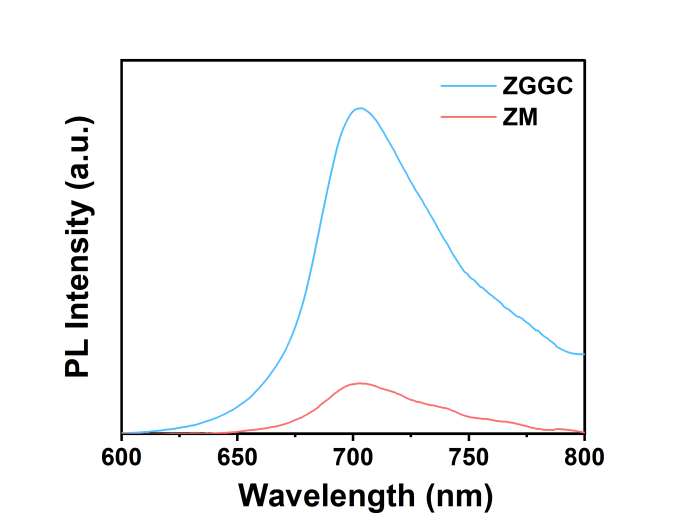


**Fig. S5.** Afterglow emission spectra of ZGGC and ZM. (excitation at 659 nm)


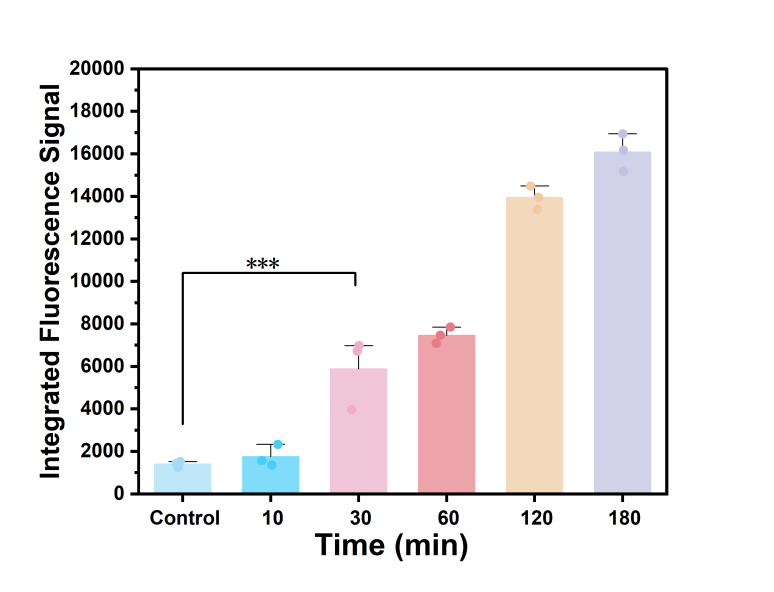


**Fig. S6.** Quantitative analysis of autophagosome fluorescence signals based on Figure 4b (mean ± SD, n=3, ****p*＜0.001).

**
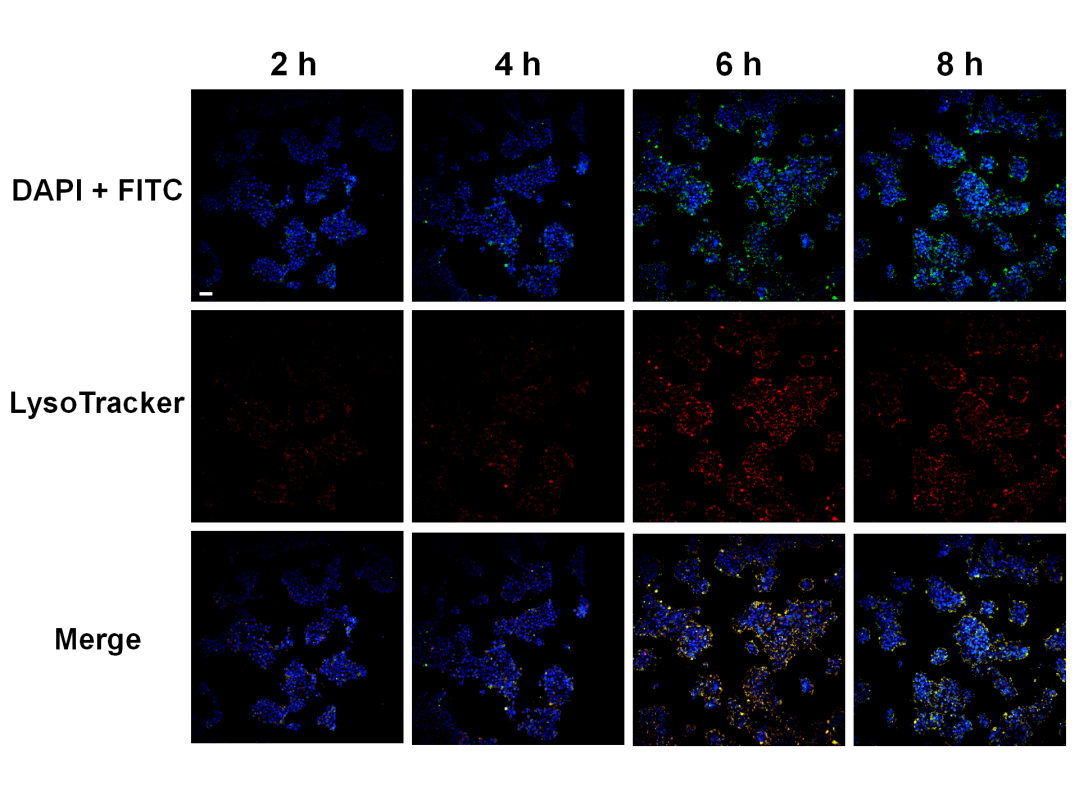
**

**Fig. S7.** Confocal fluorescence images showing FaDu cells after incubation with ZMC for 2, 4, 6, and 8 hours (ZMC: 100 µg·mL⁻¹; scale bar: 20 µm).

**
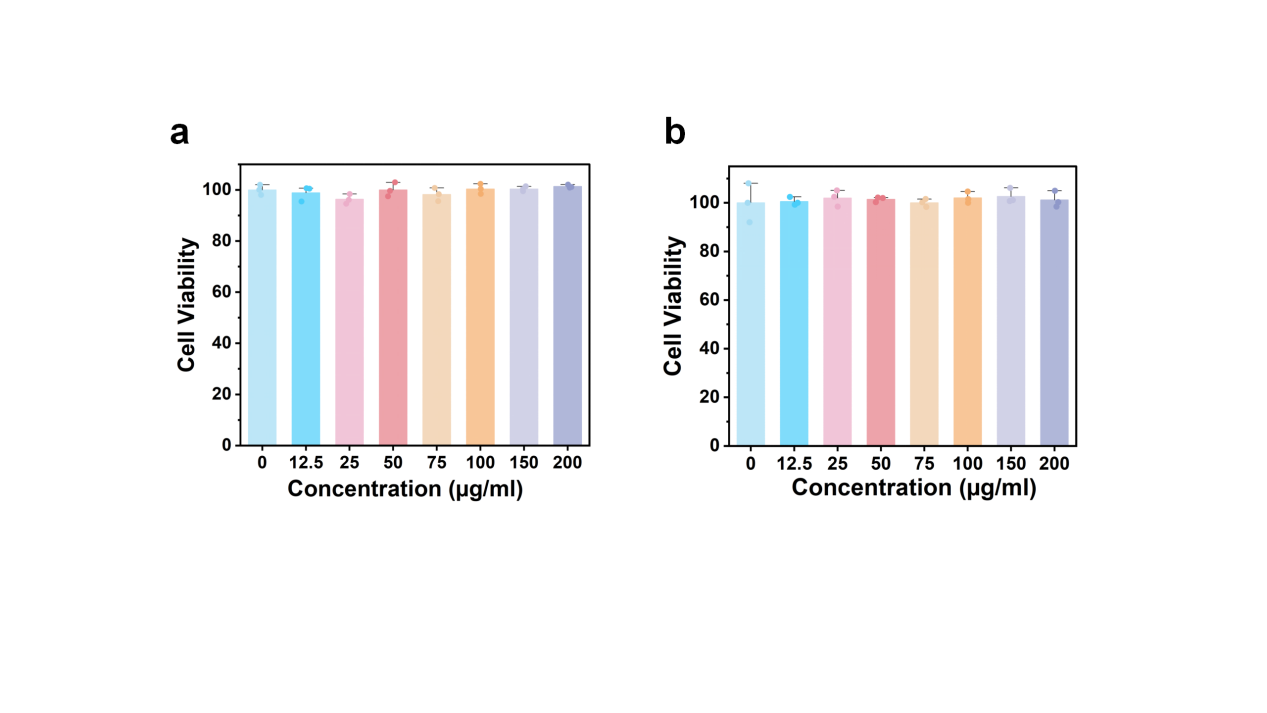
**

**Fig. S8.** Cell viability of FADU cells (a) and AML12 cells (b) treated with various concentrations of ZM (mean ± SD, n=3).

**
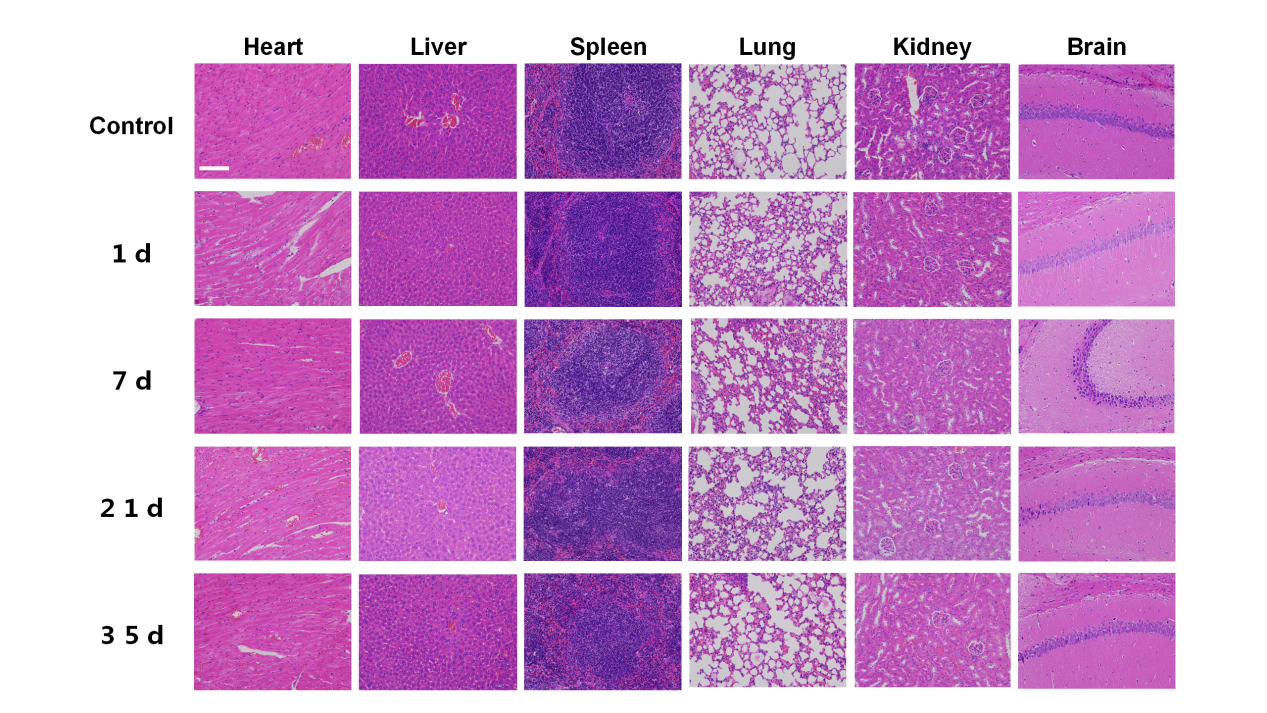
**

**Fig. S9.** Histopathological analysis (HE staining) of major organs (heart, liver, spleen, lung, kidney, and brain) from mice at different time points (scale bar:100µm).


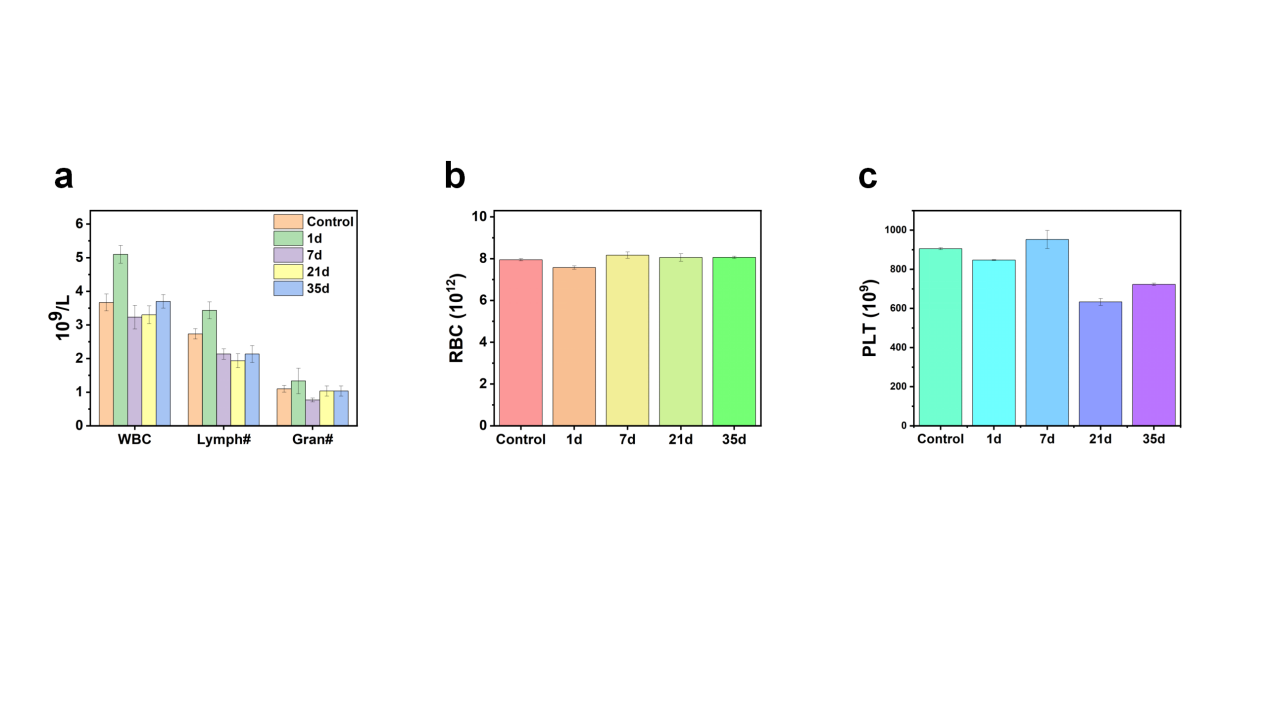


**Fig. S10.** Hematological analysis of mouse blood at various time points (a-c).


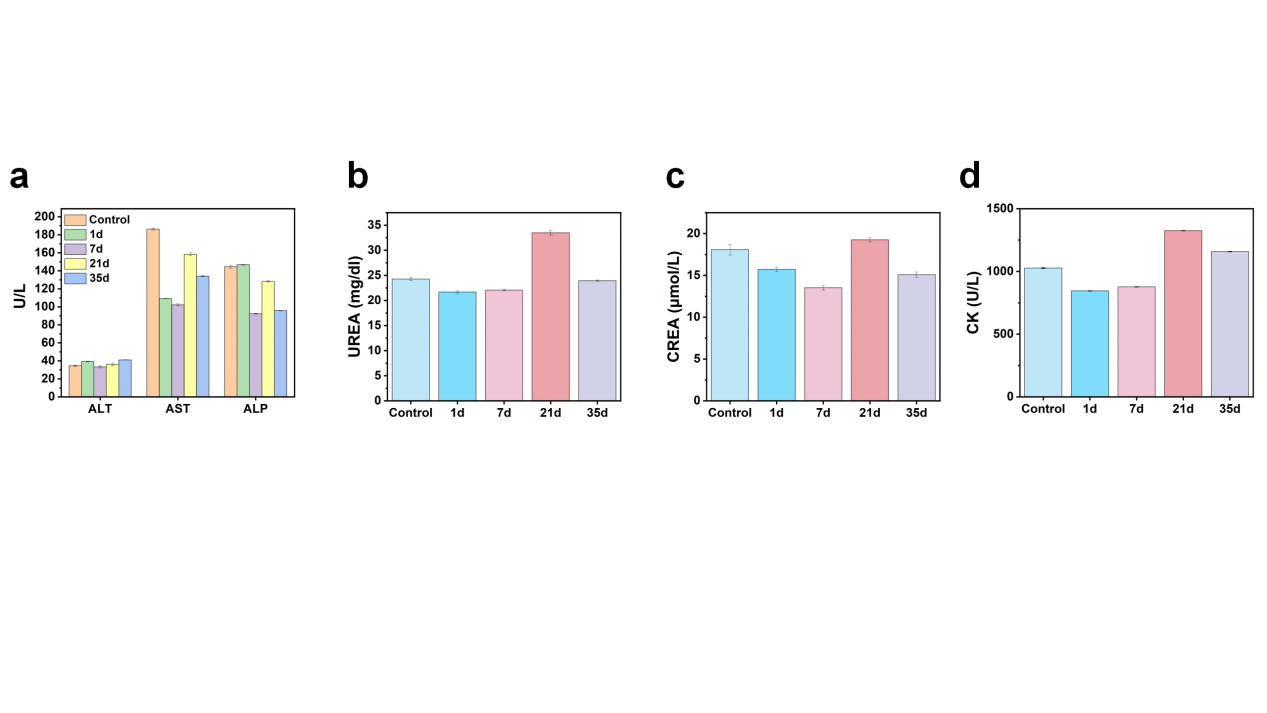


**Fig. S11.** Serum biochemical analysis in mice at various time points (a-d).​

**
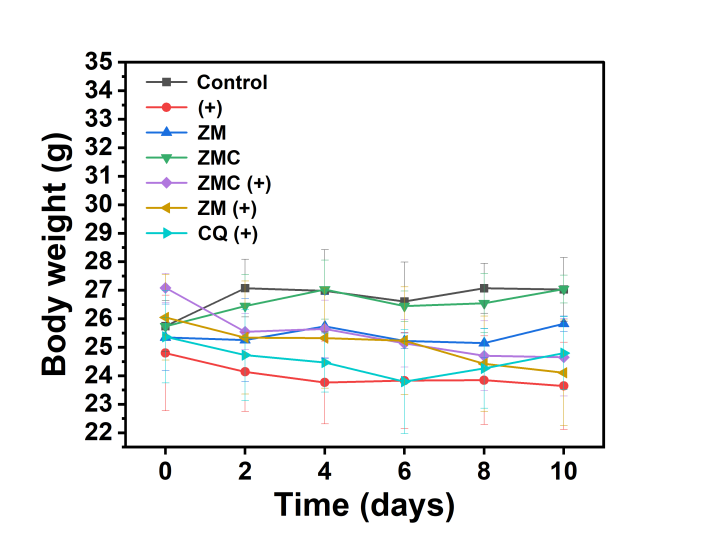
**

**Fig. S12.** Changes in body weight during the treatment period (mean ± SD, n=3, “+”: radiotherapy)


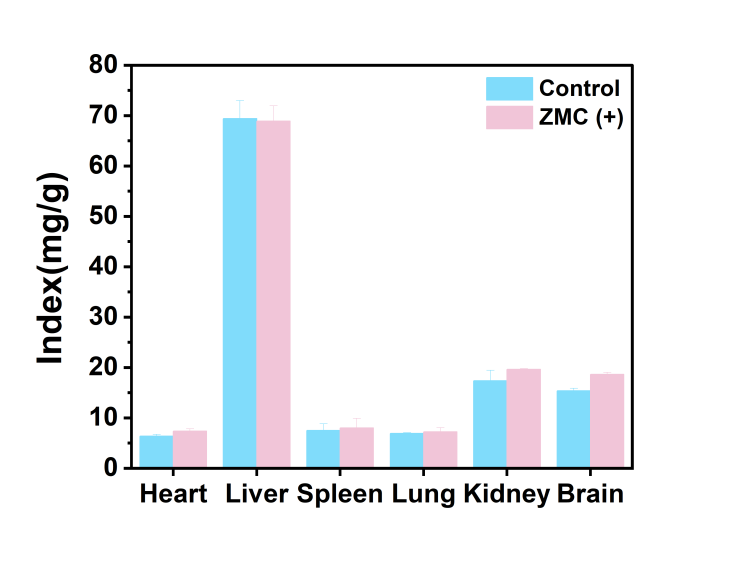


**Fig. S13.** Changes in organ coefficients of the treatment and control groups (mean ± SD, n=3, “+”:

radiotherapy).


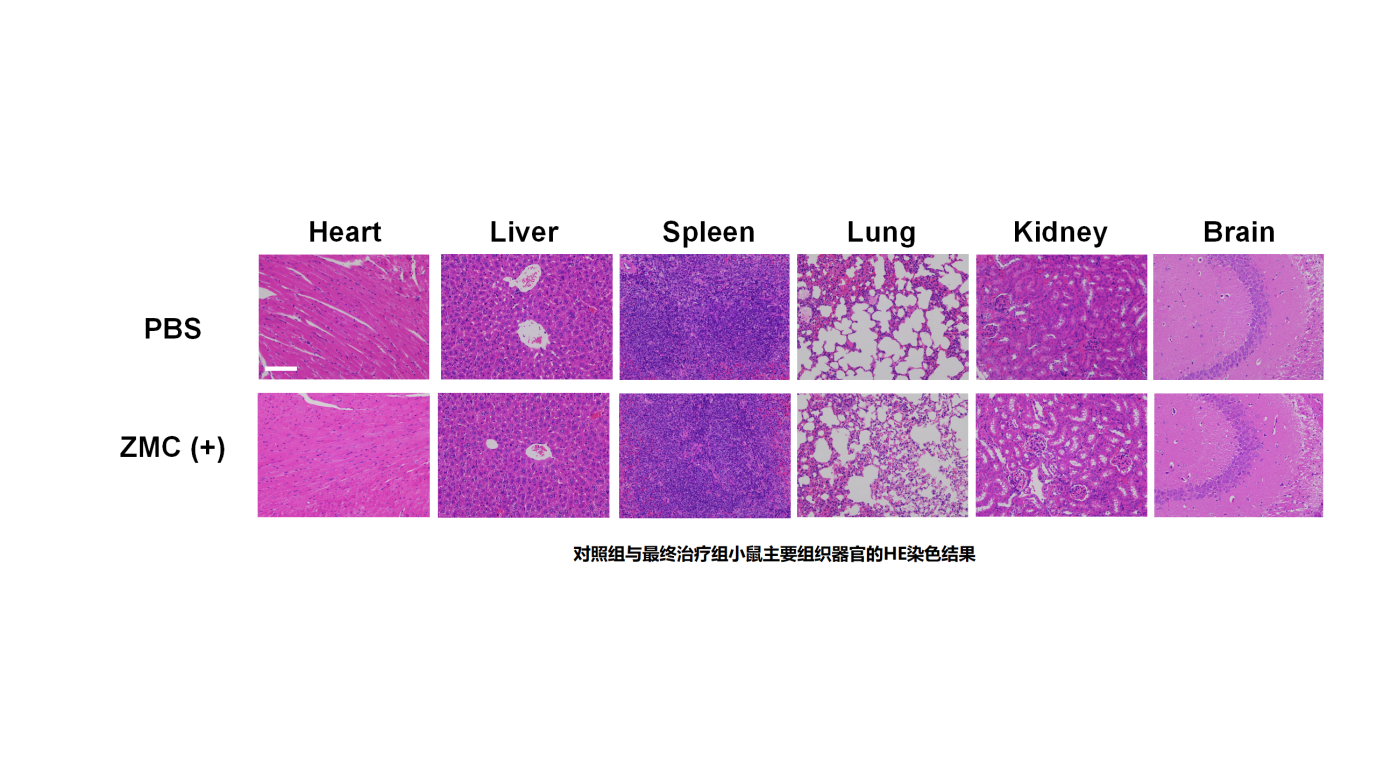


**Fig. S14.** HE staining of major organs from control and treatment groups (scale bar: 100 μm. “+”:

radiotherapy).​

**Table S1.** Surface area, pore volume, and pore size of ZGGC and ZM.​​

| **Samples** | **S_BET_**  **(m^2^∙g^-1^)** | **Pore volume**  **(cm^3^∙g^-1^)** | **Average**  **Pore size**  **(nm)** | **Most fregent pore diameter**  **(nm)** |
| --- | --- | --- | --- | --- |
| ZGGC | 77.668 | 0.304 | 15.494 | 17.396 |
| ZM | 940.072 | 1.273 | 4.319 | 7.838 |

**Table S2.**  Group treatment of mice. “√” refer to “with this treatment”

|  | **PBS (+)** | **ZM** | **ZMC** | **ZMC (+)** | **ZM (+)** | **CQ (+)** |
| --- | --- | --- | --- | --- | --- | --- |
| ZM |  | √ |  |  | √ |  |
| ZMC |  |  | √ | √ |  |  |
| CQ |  |  |  |  |  | √ |
| X-ray | √ |  |  | √ | √ | √ |
